# Replicative bypass studies of **l**-deoxyribonucleosides in Vitro and in *E. coli* cell

**DOI:** 10.1038/s41598-022-24802-5

**Published:** 2022-12-07

**Authors:** Yuhe Kan, Zhaoyang Jin, Yongqi Ke, Dao Lin, Liang Yan, Li Wu, Yujian He

**Affiliations:** 1 School of Chemical Sciences, University of Chinese Academy of Sciences, Beijing, 100049 People’s Republic of China; 2 State Key Laboratory of Natural and Biomimetic Drugs, School of Pharmaceutical Sciences, Peking University, Beijing, 100191 People’s Republic of China; 3 School of Future Technology, University of Chinese Academy of Sciences, Beijing, 100049 People’s Republic of China; 4 School of Life Sciences, Inner Mongolia University, Hohhot, 010021 Inner Mongolia People’s Republic of China; 5 Qilu Pharmaceutical (Inner Mongolia) CO., LTD., Hohhot, 010080 Inner Mongolia People’s Republic of China

**Keywords:** Biochemistry, Chemical biology

## Abstract

l-nucleosides were the most important antiviral lead compounds because they can inhibit viral DNA polymerase and DNA synthesis of many viruses, whereas they may lead to mutations in DNA replication and cause genomic instability. In this study, we reported the replicative bypass of l-deoxynucleosides in recombinant DNA by restriction enzyme–mediated assays to examine their impact on DNA replication in vitro and in *E. coli* cells. The results showed that a template l-dC inhibited Taq DNA polymerase reaction, whereas it can be bypassed by Vent (exo^-^) DNA polymerase as well as in cell replication, inserting correct nucleotides opposite l-dC. l-dG can be bypassed by Taq DNA polymerase and in *E. coli* cells, maintaining insertion of correct incoming nucleotides, and l-dG induced mutagenic replication by Vent (exo^-^) DNA polymerase. In contrast, l-dA can induced mutagenic replication in vitro and in *E. coli* cells. MD simulations were performed to investigate how DNA polymerase affected replicative bypass and mutations when d-nucleosides replaced with l-nucleosides. This study will provide a basis for the ability to assess the cytotoxic and mutagenic properties of the l-nucleoside drugs.

## Introduction

Commonly, d-nucleotides were the basic building blocks of natural nucleic acids in all known forms of life. To our knowledge, genetic polymers (DNA/RNA) with the same chirality were functional as templates in replication, transcription and translation. DNA polymerase composed of l-amino acids has strict chiral selectivity for d-deoxynucleotides. However, studies have reported that the template-directed polymerization of DNA can be catalysed by a chemically synthesized d-amino acid polymerase on an l-DNA template^1^. In addition, amount studies indicated modified or chiral nucleotides can be incorporated into DNA catalysed by various polymerases^2–7^. For example, l-nucleosides can be functioned as specific substrates for herpes simplex 1 (HSV1) thymidine kinase (TK), but not human TK^8^. Next, phosphorylated nucleoside was recognized by viral polymerases, which may provide clear clues for designing safe and effective antiviral drugs. l-nucleosides, such as *β*-l-thymidine (l–T, telbivudine), *β*-l-2′-deoxycytidine (l-dC) and β-l-2′-deoxyadenosine (l-dA) had most potent, selective and specific antiviral activity against HBV during nucleic acid synthesis^9,10^. Although l-nucleosides can be recognized by viral enzymes rather than host (bacterial or human) enzymes, once l-nucleosides were recognized by host translesion synthetase (i.e. DNA polymerase IV and V for bacterial or DNA polymerase ζ, κ, η, and ι for human) and incorporated into DNA, the tautomeric equilibrium was re-established, which might bring about cytotoxic and mutagenic effects. Wherein, l-nucleosides could be regarded as chemical mutagen leading to DNA damage.

Although various DNA repair systems (such as BER, NER, TLS, MMR, SSBR, HR, NHEJ and DNA interstrand crosslink repair pathway) were to independently or synergistically repair damaged DNA^11^, erroneous DNA repair resulted in mutations or chromosomal aberrations^12^. Additionally, when the DNA strand encounters damage during replication and the replication steps, the translesion synthesis (TLS) polymerases in cells can bypass DNA lesions to continue DNA replication. However, it might led to the mutation^13^. Fatal gene mutations caused by DNA damage have attracted more and more attention. For example, 5-Guanidino-4-nitroimidazole (NI) induced mainly G → T, G → A and some G → C mutation and strongly blocked replication in *E. coli* cells^14^. Two strong mutagenic agents, stereoisomers of spiroiminodihydantoin (Sp1 and Sp2), caused mainly G → C (72% for Sp1 and 57% for Sp2) and G → T (27% for Sp1 and 41% for Sp2) mutations with strong replication inhibition (91%)^15^. 3-methyldeoxycytidine (m3C) and 3-ethyldeoxycytidine (e3C) were 30% mutagenic in SOS^-^ cells, but rising to 70% in SOS^+^ cells^16^. Aflatoxin B1-N7-dG adduct (AFB1-N7-dG) caused mutation frequency of 4%, which dominantly give rise to G → T mutation^17^. *O*^4^-alkyldT can induce substantial frequencies T → C transition mutation and moderately inhibited DNA replication with the bypass efficiencies ranging from 20 to 33% in HEK293T cells ^18^. Previously, we reported that l-thymidine induced self-repair by B family polymerases ^19^ whereas l–T → dA (13%), T (22%), dG (19%) and dC (46%) mutations in *E. coli* cells^20,21^. The results inspire us to explore the fidelity and cytotoxicity for l-dC, l-dG and l-dA during replication, facilitating understanding of the chiral transfer in the process of nucleic acid evolution.

In the current study, the recombinant pUC19 vector containing l-dA, l-dG and l-dC were constructed and subsequently transfected into *E. coli* cells. The replicative bypass of l-dC, l-dG and l-dA in recombinant DNA in vitro and in *E. coli* cells was to evaluate the mutagenic effect of l-deoxynucleotides during DNA replication using restriction enzyme–mediated assays^20,21^. The result showed that the bypass efficiency of l-nucleosides in *E. coli* cells was relatively high compared to Taq DNA polymerases or Vent (exo^-^) DNA polymerases, and even the replicative bypass of l-dC was completely inhibited by Taq DNA polymerase. In addition, l-dA induced more mutation types in cells, including purine → purine conversion and purine → pyrimidine transversion. This was similar to our previously reported results for l–T in *E. coli* cells replication. However, l-dG did not cause a mismatch in *E. coli* cells but did mutate with Vent (exo^-^) DNA polymerases. Effect of l-nucleosides on replicative bypass and mutations with DNA polymerase were further investigated by using MD simulations. Our study will provide a basis for the ability to assess the cytotoxic and mutagenic properties of the l-nucleoside drugs.

## Results

### Construction of **l**-deoxynucleoside-bearing plasmids for DNA replication

**Figure 1:**
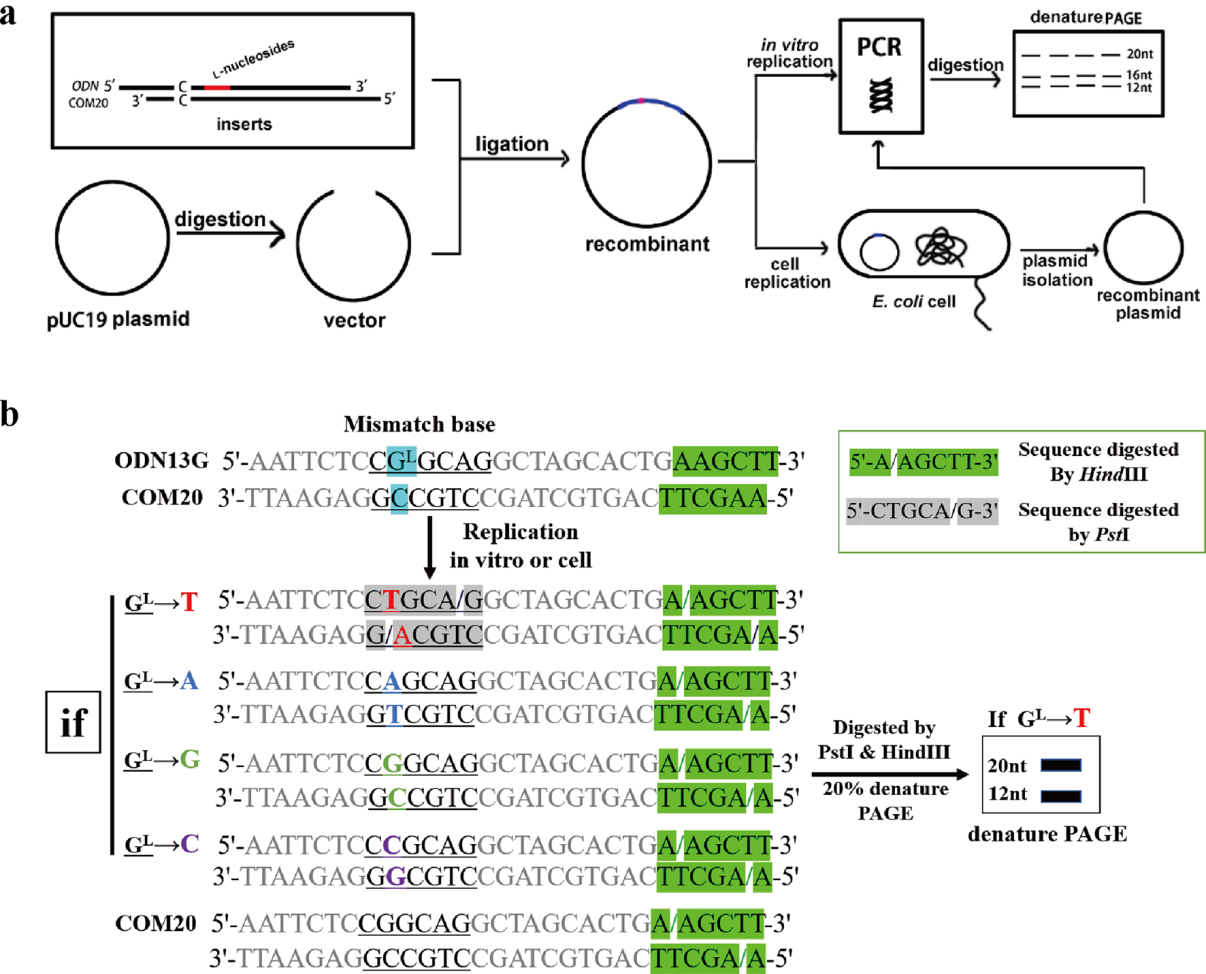
Schematic diagram of restriction endonuclease mediated assay for study on replication of DNA containing lesion.

The aim of this study was to elucidate comprehensively the impact of l-nucleosides on the bypass and fidelity of DNA replication in vitro and in *E. coli* cells. To this end, we first synthesized a series of site-specific l-nucleoside-carrying ODNs for each type of l-nucleosides and a complement strand COM20 (Table 1). For example, nucleoside T, dG, dC, dA on *Pst*I recognition sequence within STD strand ($5{^\prime}\hbox{-AATTCTC}\underline{\rm CTGCAG}\hbox{GCTAGCACTGA-}3{^\prime}$) was substituted by l-dG, respectively, of which “lesion-containing strands” were named G13 ($5{^\prime}\hbox{-AATTCTC}\underline{{\rm CG}{^{\rm L}}{\rm GCAG}}\hbox{GCTAGCACTGA-}3{^\prime}$), G14 ($5{^\prime}\hbox{-AATTCTC}\underline{{\rm CTG}{^{\rm L}}{\rm CAG}}\hbox{GCTAGCACTGA-}3{^\prime}$), G15 ($5{^\prime}\hbox{-AATTCTC}\underline{{\rm CTGG}{^{\rm L}}{\rm AG}}\hbox{GCTAGCACTGA-}3{^\prime}$) and G16 ($5{^\prime}\hbox{-AATTCTC}\underline{{\rm CTGCG}{^{\rm L}}{\rm G}}\hbox{GCTAGCACTGA-}3{^\prime}$). Then, the complement strand (COM20) was annealing with lesion-containing strands to give original lesion-containing double strand, and a C:C mismatch was inserted around the recognition site of *Pst*I of the double strand, which was to distinguish the replication products of lesion-containing strands from the natural strands as noted previously^21^. To assess the bypass efficiencies and mutation frequencies of the l-nucleosides, we ligated the aforementioned inserts containing l-nucleosides into pUC19 genome and performed a restriction enzyme–mediated assays to examine how these lesions inhibit DNA replication and induce mutations in vitro and in *E. coli* cells (Fig. 1). Prior to assessing the cytotoxicity and fidelity induced by l-nucleosides, the digestion products corresponding to 24-mer ODNs cleaved by *EcoR*I and *Hind*III restriction endonuclease were detected, indicating successful construction of l-nucleosides-containing genomes during ligation process. Then, we employed two restriction enzymes, i.e. *Pst*I and *Hind*III, to digest the PCR products of the progeny genome, affording 20mer ODN fragments from the lesion-containing or control genome (STD) (Fig. 1). The released ODNs were subjected to denature PAGE analyses to identify the replication products, as described elsewhere^21^. As illustrated in Fig. 1, in this vein, if the correct nucleotide opposite l-nucleosides was incorporated during DNA replication, which restore *Pst*I recognition sequence. *Pst*I restriction enzyme was allowed for the selective digestion of the progeny genomes emanating from the replication of the strand that initially contained the l-nucleosides due to C:C mismatch^21^. The complementary opposite strand (COM20, 5′-CTGCAC-3′) in a recombinant vector produced a strand of 3′-CACGTC-5′/5′-CTGCAC-3′ in replication, which is different from restriction digestion fragments of 3′-GACGTC-5′/5′-CTGCAG-3′ formed from lesion-containing strand if a corrected nucleotide was incorporated opposite the lesion site. The amplified strand from COM20 could not be cleaved by *PstI* and thus was not detected by analyzing 20mer bands in the gel. In this respect, sequential digestion of l-nucleoside-containing strands with *Hind*III and *Pst*I would give a 20mer fragment (5′-AGCTTCAGTGCTAGCCTGCA-3′) on 20% denature PAGE gel if incorporation of nucleotides opposite l-nucleoside restored the *Pst*I recognition sequence. On the contrary, no band was present corresponding to 20mer fragment due to disrupting *Pst*I recognition sequence. To take a concrete example, replication products (5′-CTGCAG-3′) were found to be further digested by *Pst*I when dA was incorporated opposite l-dG within G13 (5′-CG^L^GCAG-3′) during DNA replication. However, the corresponding product harboring an l-dG → dC, dA or dG mutation at the lesion site, i.e. 5′-CCGCAG-3′, 5′-CAGCAG-3′ or 5′-CGGCAG-3′, could not be digested by *Pst*I. Similarly, for G14 (5′-CTG^L^CAG-3′), G15 (5′-CTGG^L^AG-3′) and G16 (5′-CTGCG^L^G-3′), restriction digestion would occur when only dC, dG and T were incorporated opposite l-dG, respectively. Thus, with the combination of the two enzyme digestion procedures, we were able to distinguish unequivocally the four potential types of replication products, and by monitoring the products from the strand of 20mer ODNs (5′-AGCTTCAGTGCTAGCCTGCA-3′), we could quantify the bypass efficiency and mutation frequencies.

**Table 1:**
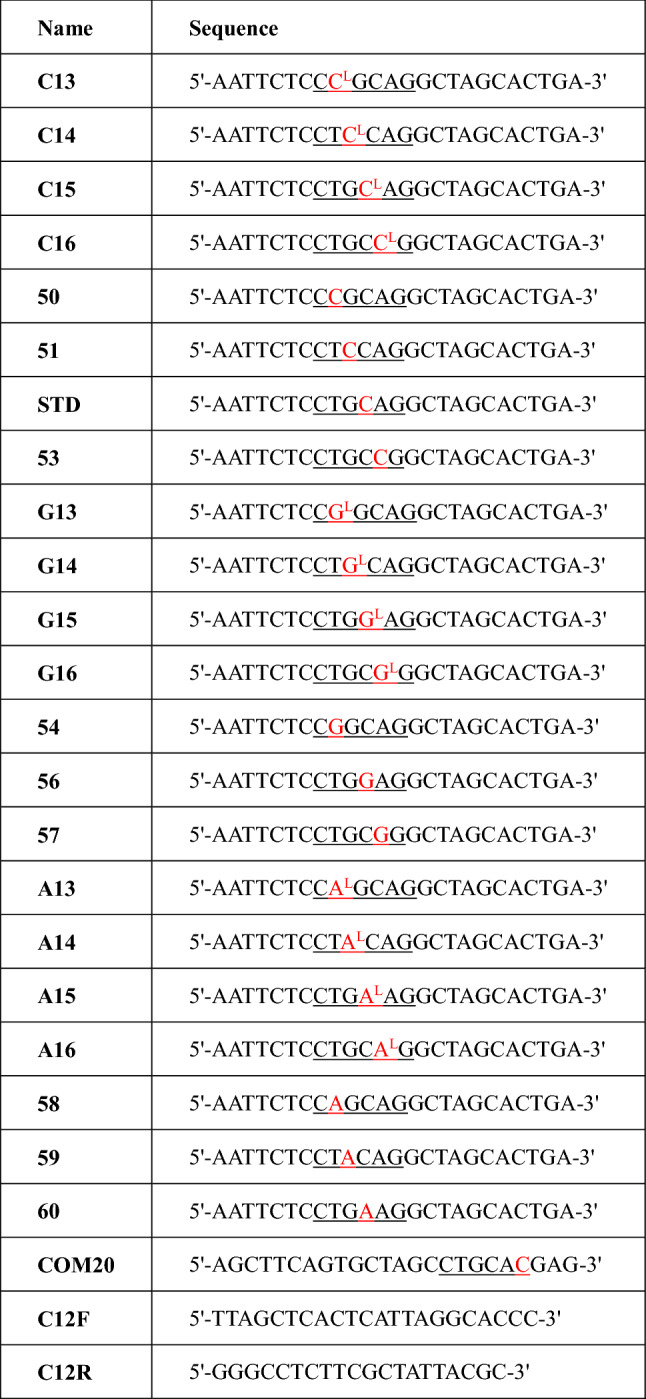
Single-strand oligonucleotides used in this study.

Underlined sequences : nucleoside (red site) of *PstI* recognition site was replaced by l-nucleosides and natural nucleosides, respectively.

The bypass efficiencies of the l-nucleosides were then calculated from the ratio of the combined intensities of bands observed for the 20mer products from the lesion-containing genome over the intensity of the 20mer product from the control genome STD. With the use of this method, we were able to determine quantitatively the degrees to which the l-dC, l-dG and l-dA lesions inhibit DNA replication and induce mutations in *E. coli* cells. Although *Pst*I-digestion can be conducted for pUC19 self-ligation plasmid, only 16-mer band on PAGE gel will not interfere assessment of incorporation frequency and bypass efficiency.

The bypass efficiencies and mutation frequency were assessed by our previous method^21^. Simply, the bypass efficiencies for the l-nucleoside-carrying genomes were then normalized against that for the control genome. Then, the bypass efficiencies of the l-nucleoside were calculated by comparing intensities of 20mer bands observed from the l-nucleoside-containing genome over the intensity of the control genome. The mutation frequency of l-nucleosides was obtained based on above bypass efficiencies, calculating by ratio each bypass efficiency (i.e. l-dG → T) to total bypass efficiency, i.e*.* l-dG → T, dG, dC and dA. Thus, by monitoring the products and evaluating bypass efficiency and mutation frequency from the replication of l-nucleosides-containing strand, we were able to study the effect of l-nucleosides on DNA replication in vitro and in cells^20,21^.

*E. coli* cells are the most widely studied bacteria that live in the intestines of people and animals and contain five types of DNA polymerases contributing DNA replication. DNA polymerase I is the most abundant and high-fidelity polymerase. Certainly, DNA polymerase III (family C) is also the major replicase^22^. And DNA polymerase II has 3′-5′ nucleic acid exonuclease activity and restarts replication after it has been halted by DNA strand damage. DNA polymerase I and DNA polymerase II belongs to A and B family polymerases, respectively^23^. It was reported that A family and B family polymerases had significantly different effect during DNA replication^19^ which inspired us to investigate replicative bypass of l-deoxyribonucleosides using A and B family polymerases. Due to commercial availability, Taq DNA polymerase and Vent (exo^-^) DNA polymerase, which respectively represented A and B family polymerases, were used for in vitro DNA replication.

Previously, we constructed four l–T-containing recombinants using T13 (5′-CT^L^GCAG-3′), T14 (5′-CTT^L^CAG-3′), T15 (5′-CTGT^L^AG-3′) and T16 (5′-CTGCT^L^G-3′) to assess the bypass efficiencies and mutation frequencies of the l–T lesions in vitro and in cells. By a restriction enzyme-mediated assay provided by us, we showed the l–T was bypassed in *E. coli* cells (99%), and the bypass efficiency in cells was higher than Taq DNA polymerases (71%) and Vent (exo^-^) DNA polymerases (47%). In addition, the cell replication across l–T lead to l–T → dA (13%), T (22%), dG (19%) and dC (46%) mutations^21^. Next, we hope to gain a comprehensive understanding of the impact of l-deoxynucleotides on DNA replication in vitro and in *E. coli* cells.

### Impacts of l-dC on the bypass efficiency and mutation frequency of DNA replication in vitro and in *E. coli* cells

To understand the impact of l-dC lesions on DNA replication in vitro and in *E. coli* cells, we constructed four l-dC-containing recombinants using C13 (5′-CC^L^GCAG-3′), C14 (5′-CTC^L^CAG-3′), C15 (5′-CTGC^L^AG-3′) and C16 (5′-CTGCC^L^G-3′) to assess the bypass efficiencies and mutation frequencies of the l-dC lesions. In this assay, DNA containing l-nucleosides was directly replicated by Taq DNA polymerase and Vent (exo^-^) DNA polymerases for in vitro study. For study of cell replication, the recombinants containing l-nucleosides were transformed into *E. coli* cells, following by PAGE analysis for digested PCR products of plasmid genome.

**Figure 2:**
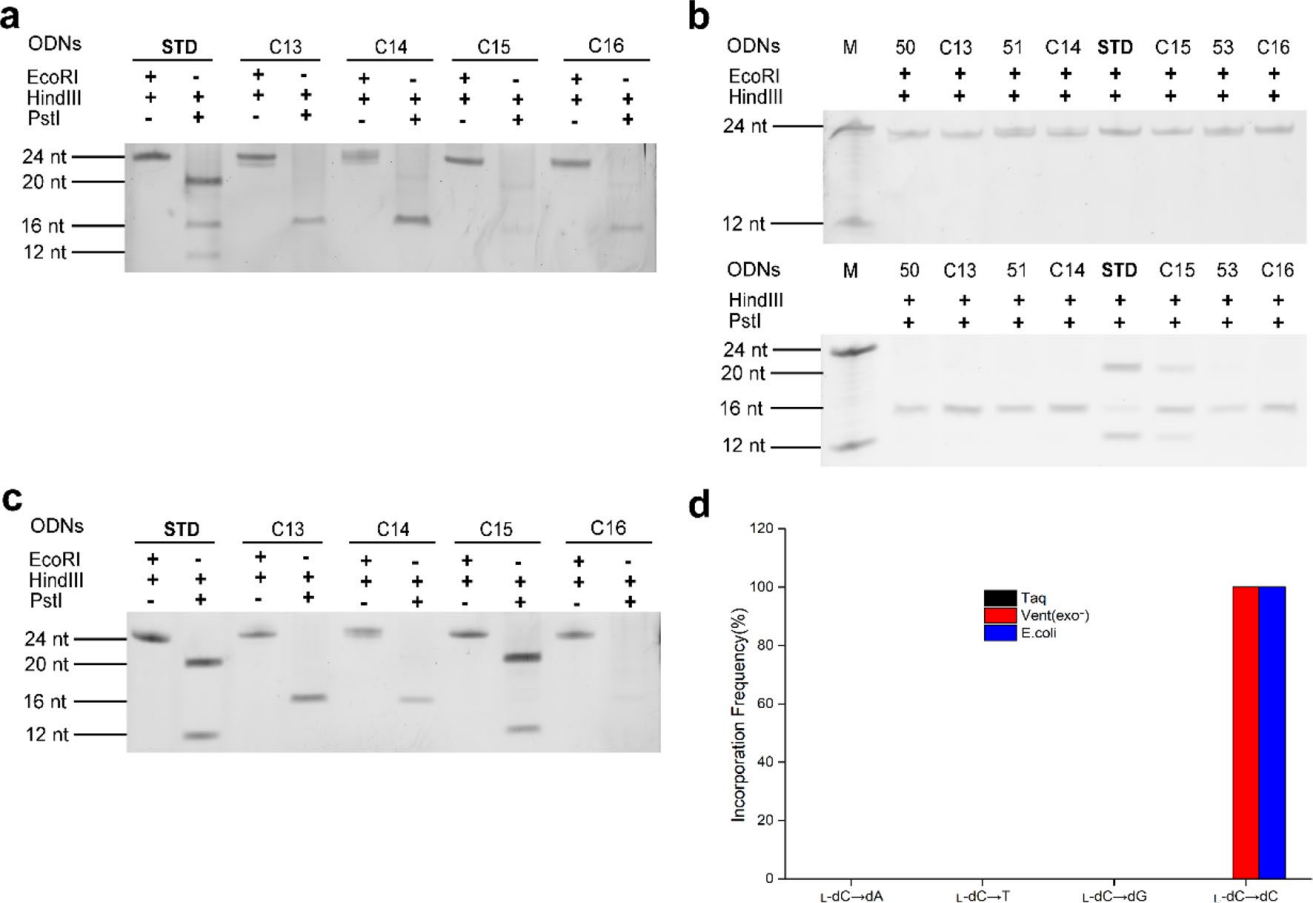
PAGE analysis of restriction fragments of replication products arising from the l-dC lesion-bearing genome (**a**) Taq DNA polymerase (**b**) Vent (exo^-^) DNA polymerase (**c**) *E. coli* cells and (**d**) its mutation frequency.

Two restriction enzymes, *Hind*III and *EcoR*I, were employed to digest replication products in vitro and in cells from four lesion-containing genomes, only 24mer bands was observed on denature PAGE gel, which indicated successful construction of l-C-bearing plasmids (Fig. 2). In addition, PCR products from recombinants containing l-nucleosides were digested with *EcoR*I and *Hind*III, no other bands (> 24-mer or < 24-mer ODNs) were observed on PAGE gel, which indicated no nucleotide insertion and deletions either in vitro or in *E. coli* cells.

The DNA fragments corresponding to 16-mer restriction products digested by *Hind*III and *Pst*I were detectable, likely due to fragment generation by the self-ligated pUC19 plasmid. As expected, PCR products of STD (Fig. 2a (Fig. S1), lane 2; Fig. 2b (Fig. S2), lane 6 and Fig. 2c (Fig. S3), lane 2) were digested by *Pst*I and *Hind*III to produce 20mer fragments. Because the *Pst*I digestion recognition sequence within insert sequence was not scrambled, so it can be digested by *Pst*I and *Hind*III to generate products corresponding to 20mer fragments. In contrast, *Pst*I recognition sequence with one nucleoside substituted (50, 51 and 53) will not be digested by *Pst*I, which can't produce 20mer ODNs (Fig. 2b (Fig. S2), lane 2, 4 and 8) on PAGE gel due to disorder of its *Pst*I recognition sequence.

Figure 2a (Fig. S1) showed that replication products from recombinants, of which T, dG, dC and dA on STD (5′-CTGCAG-3′) was replaced by l-dC, was digested by *Pst*I and *Hind*III under catalysis of Taq DNA polymerase. If dA, dC, dG and T were incorporated opposite l-dC of C13, C14, C15 and C16, respectively, its replication products would be digested by *Pst*I due to restores of its recognition sequences, and thus 20mer band on PAGE gel was observed. Actually, no 20mer fragment was detectable on gel for all the above experimental groups (Fig. 2a (Fig. S1), lane 4, 6, 8 and 10), showing dA, dC, dG and T was not incorporated opposite l-dC during DNA replication. These results indicated that the replication of DNA containing l-dC was inhibited by Taq DNA polymerase.

Different from what happened on Taq DNA polymerase, a 20mer oligonucleotide was observed for C15 (Fig. 2b (Fig. S2), lane 7) when catalyzed by Vent (exo^−^) DNA polymerase, which indicated l-dC paired with dG in replication products. No bands corresponding to 20mer fragments were present for C13, C14 and C16 (Fig. 2b (Fig. S2), lane 3, 5 and 9), showing no dA, dC and T was incorporated opposite l-dC during DNA replication. Prior to normalizing band intensities of the 20mer fragment to that of the 24-mer fragment, the bypass efficiencies of the l*-*dC lesions were then calculated from the ratio of the combined intensities of bands observed for the 20mer products from the lesion-containing genome over the intensity of the 20mer product from the control genome (STD). And incorporation frequencies were calculated from the percentage of a mutagenic product relative to all possible products from the control genome. The bypass efficiency of 39% was obtained (Supplementary Table S1 and S2) and l-dC induced no mutagenic replication catalysed by Vent (exo^-^) DNA polymerase.

For replication in *E. coli* cells, the result was same as in vitro replication catalysed by Vent (exo^-^) DNA polymerase. Only one band corresponding to 20mer fragment was obtained for C15 (Fig. 2c (Fig. S3), lane 8), which indicated l-dC: dG pairing formed in *E. coli* cells, with bypass efficiency being 81% (Supplementary Table S1 and S2). No bands corresponding to 20mer fragment were present for C13, C14 and C16 (Fig. 2c (Fig. S3), lane 4, 6 and 10), showing no dA, dC and T was incorporated opposite l-dC in cell replication.

Hence, these results revealed that l-dC completely block the DNA replication machinery under catalysis of Taq DNA polymerases, whereas l-dC can be bypassed by Vent (exo^-^) DNA polymerases or in *E. coli* cells. The bypass efficiency in *E. coli* cells (81%) was higher than that obtained under catalysis of Vent (exo^-^) DNA polymerases (39%). What's more, no mutagenic replication was found for l-dC-containing DNA in vitro and in *E. coli* cells.

### Impacts of _L_-dG on the bypass efficiency and mutation frequency of DNA replication in vitro and in *E. coli* cells

To assess the bypass efficiencies and mutation frequencies of the l-dG lesions, four l-G-containing recombinants were parallelly constructed by incorporation of G13 (5′-CG^L^GCAG-3′), G14 (5′-CTG^L^CAG-3′), G15 (5′-CTGG^L^AG-3′) and G16 (5′-CTGCG^L^G-3′).

**Figure 3:**
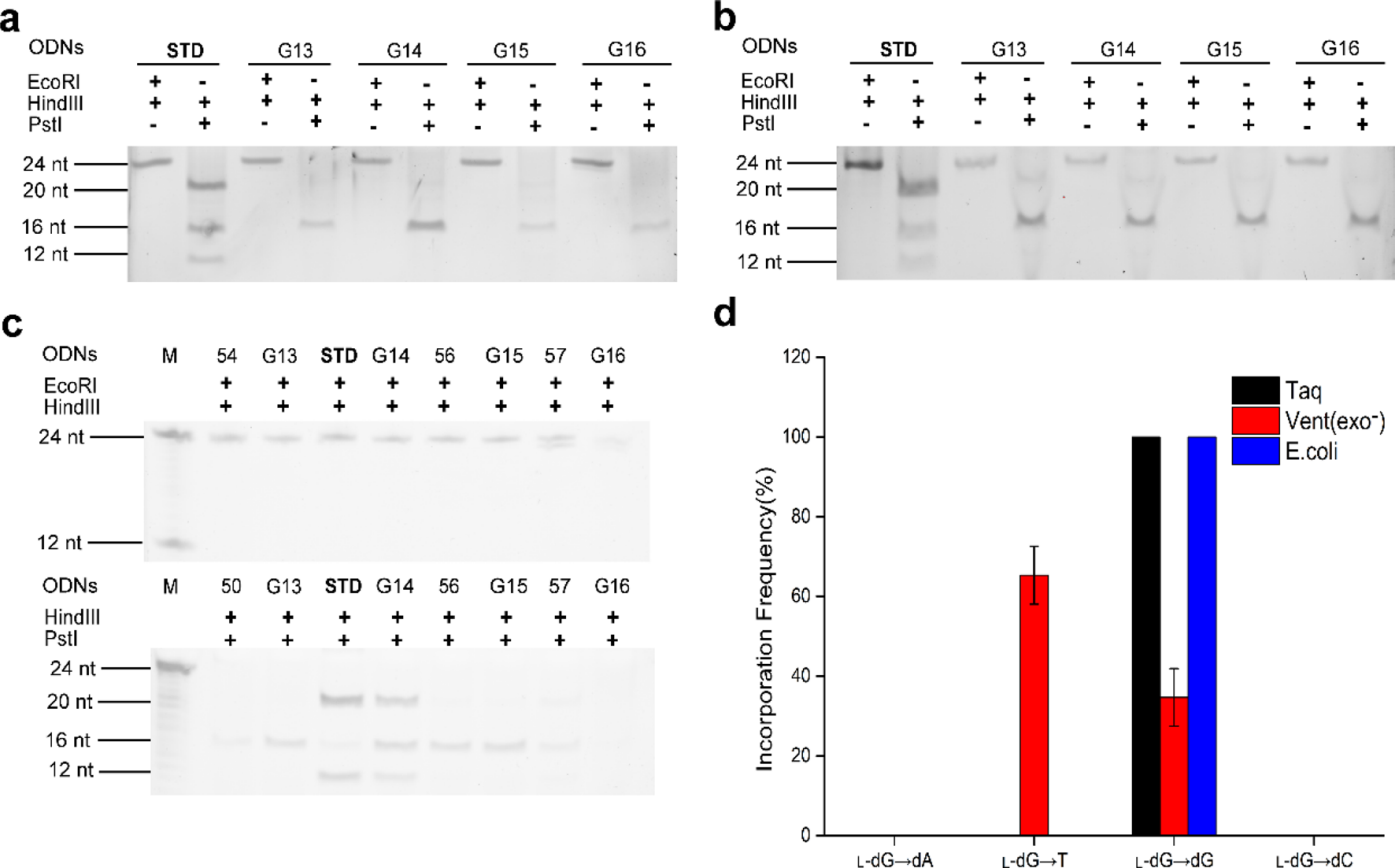
PAGE analysis of restriction fragments of replication products arising from the l-dG lesion-bearing genome (**a**) Taq DNA polymerase (**b**) Vent (exo^-^) DNA polymerase (**c**) *E. coli* cells and (**d**) its mutation frequency.

As shown in Fig. 3, 24mer bands on denature PAGE gel corresponding to the digested products by *Hind*III and *EcoR*I were present, indicating successful construction of l-G-bearing plasmids. In addition, undesirable 16-mer ODNs from *Pst*I and *Hind*III digestion was observed, suggesting there was self-ligated pUC19 plasmid. For positive restriction endonuclease assay, 20mer fragments from *Pst*I and *Hind*III digested PCR products of STD (Fig. 3a (Fig. S4), lane 2; Fig. 3b (Fig. S5), lane 2 and Fig. 3c (Fig. S6), lane 4) were obtained, as expected.

However, for the reaction system catalysed by Taq DNA polymerases, a 20-mer oligonucleotide band was found for G14 (Fig. 3a (Fig. S4), lane 6) when the replication products were incubated with *Pst*I and *Hind*III, which suggested that replication of G14 with replacing G with l-dG resulted in l-dG: dC pairing. Moreover, l-dG induced l-dG → dG substitution with bypass efficiency of 19% (Supplementary Table s S1 and S2). By comparation, 20mer band was absent on gel for G13, G15 and G16 (Fig. 3a (Fig. S4), lane 4, 8 and 10), in which l-dG was substituted for T, C and A of 5′-CTGCAG-3′, respectively. The result indicated that no dA, dG and T was incorporated opposite l-dG during DNA replication. That is, l-dG did not result in a mutation in the reaction system catalysed by Taq DNA polymerases.

In the reaction system catalysed by Vent (exo^-^) DNA polymerases, 20-mer DNA fragments of digested replication products can be detected in recombinant plasmids G14, where l-dG substituted for G of 5′-CTGCAG-3′ (Fig. 3b (Fig. S5), lane 6), which indicated that dC was incorporated opposite l-dG. Additionally, 20-mer DNA fragments was observed for G13 (Fig. 3b (Fig. S5), lane 4), indicated that dA was incorporated opposite l-dG of 5′-CG^L^GCAG-3′, that is, l-dG induced l-dG → T transversion mutation besides l-dG → G. The bypass efficiency of l-dG was 12% and the mutation frequency of l-dG → T was 65% (Supplementary Table s S1 and S2). In contrast, no bands corresponding to 20mer fragments was found for G15 and G16 (Fig. 3b (Fig. S5), lane 8 and 10) proved that dG and T was not incorporated opposite l-dG during DNA replication catalysed by Vent (exo^-^) DNA polymerase.

For replication investigation in *E. coli* cells, only in the case of G14 was a 20-mer oligonucleotide band found on gel (Fig. 3a (Fig. S4), lane 6) when the replication products were incubated with *Pst*I and *Hind*III. This indicated that replication of DNA containing l-dG resulted in l-dG: dC pairing, with bypass efficiency being 82% (Fig. 3c (Fig. S6), lane 5; Supplementary Tables S1 and S2), whereas dA, dG and T was not incorporated opposite l-dG during DNA replication (Fig. 3c (Fig. S6), lane 3, 7 and 9).

Hence, for study on l-dG-containing DNA replication, Taq DNA polymerase and Vent (exo^−^) DNA polymerase were inhibited by l-dG, and the bypass efficiencies were decreased to 19% and 12%, respectively. But, in cell replication assay resulted in apparent increase in bypass efficiency (82%) relative to in vitro replication. Mutagenic replication was found in vitro but not in cells, which may attribute to repair machinery and related proteins in cells.

### Impacts of **l**-dA on the bypass efficiency and mutation frequency of DNA replication in vitro and in *E. coli* cells

**Figure 4:**
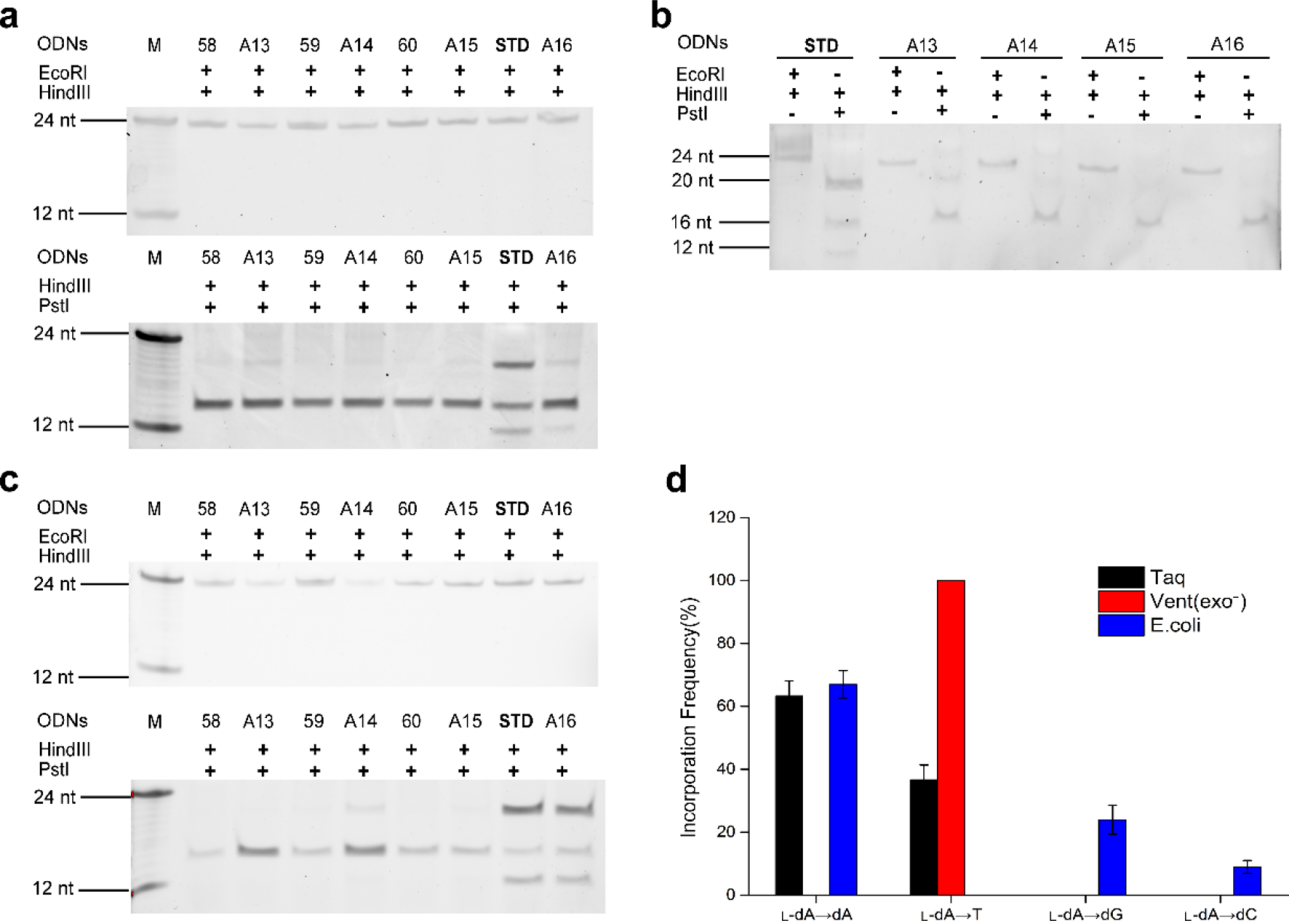
PAGE analysis of restriction fragments of replication products arising from the l-dA lesion-bearing genome (**a**) Taq DNA polymerase (**b**) Vent (exo^-^) DNA polymerase (**c**) *E. coli* cells and (**d**) its mutation frequency.

We constructed four l-dA-containing recombinants using A13 (5′-CA^L^GCAG-3′), A14 (5′-CTA^L^CAG-3′), A15 (5′-CTGA^L^AG-3′) and A16 (5′-CTGCA^L^G-3′) to assess the bypass efficiencies and mutation frequencies of the l-dA lesions. Figure 4 indicated successful l-A-bearing plasmids and self-ligated pUC19 plasmid. As expected, *Pst*I and *Hind*III digestion products corresponding to 20mer ODNs was obtained for STD (Fig. 4a (Fig. S7), lane 8; Fig. 4b (Fig. S8), lane 2 and Fig. 4c (Fig. S9), lane 8) revealed that the *Pst*I digestion recognition sequence within insert sequence was not destroyed. In contrast, no 20-mer ODNs was observed for 58, 59 and 60 (Fig. 4a (Fig. S7) and 4c (Fig. S9), lane 2, 4 and 6) which resulted from destroyed *Pst*I recognition sequence.

The effect of l-dA on DNA replicative bypass and fidelity was investigated by Taq and Vent (exo^-^) DNA polymerases as well as in cells. Figure 4a (Fig. S7) showed experimental results in the case of Taq polymerase. Replication products of A13 and A16 were digested by restriction endonuclease *Pst*I and *Hind*III to produce 20-mer fragments (Fig. 4a (Fig. S7), lane 3 and lane 9), whereas no 20mer restriction-digested PCR fragments of A14 and A15 were detected (Fig. 4a (Fig. S7), lane 5 and lane 7). This indicated that dA and T rather than dC and dG were incorporated opposite l-dA during DNA replication. Furthermore, Taq DNA polymerase bypassed l-dA with a low bypass efficiency of 16%. l-dA paired with T with frequency being 63% and induced l-dA → T transversion at a frequency of 37% (Supplementary Table s S1 and Table S2).

Different from Taq DNA polymerase, only one band corresponding to digested 20-mer DNA fragment of PCR product was detected in recombinant plasmids A13 containing l-dA lesion when catalysed by Vent (exo^-^) DNA polymerases (Fig. 4b (Fig. S8), lane 4), indicate that l-dA was strongly miscoding, and it induced completely l-dA → T transversion mutation catalysed by Vent (exo^-^) DNA polymerases, with bypass efficiency being 20% (Supplementary Tables S1 and Table S2).

In the study of the replication of DNA containing l-dA in cells, PCR products of A14, A15, and A16 were digested by restriction endonuclease *Pst*I and *Hind*III to produce 20-mer fragments (Fig. 4c (Fig. S9), lane 5, lane 7 and lane 9), verifying the l-A → dA, dG, and dC mutation occurred during cell replication, and l-dA did not induce l-dA → T mutation (Fig. 4c (Fig. S9), lane 3). In addition, the results showed that bypass efficiency of l-dA in *E. coli* cells was up to 74%, which was 3–5 times as high as that for Taq DNA polymerases (16%) and Vent (exo^-^) DNA polymerases (20%). Also, l-A → dA, dG and dC with mutation frequency being 67%, 24% and 9% (see Supplementary Table s S1and Table S2 online).

## Discussion

**Figure 5:**
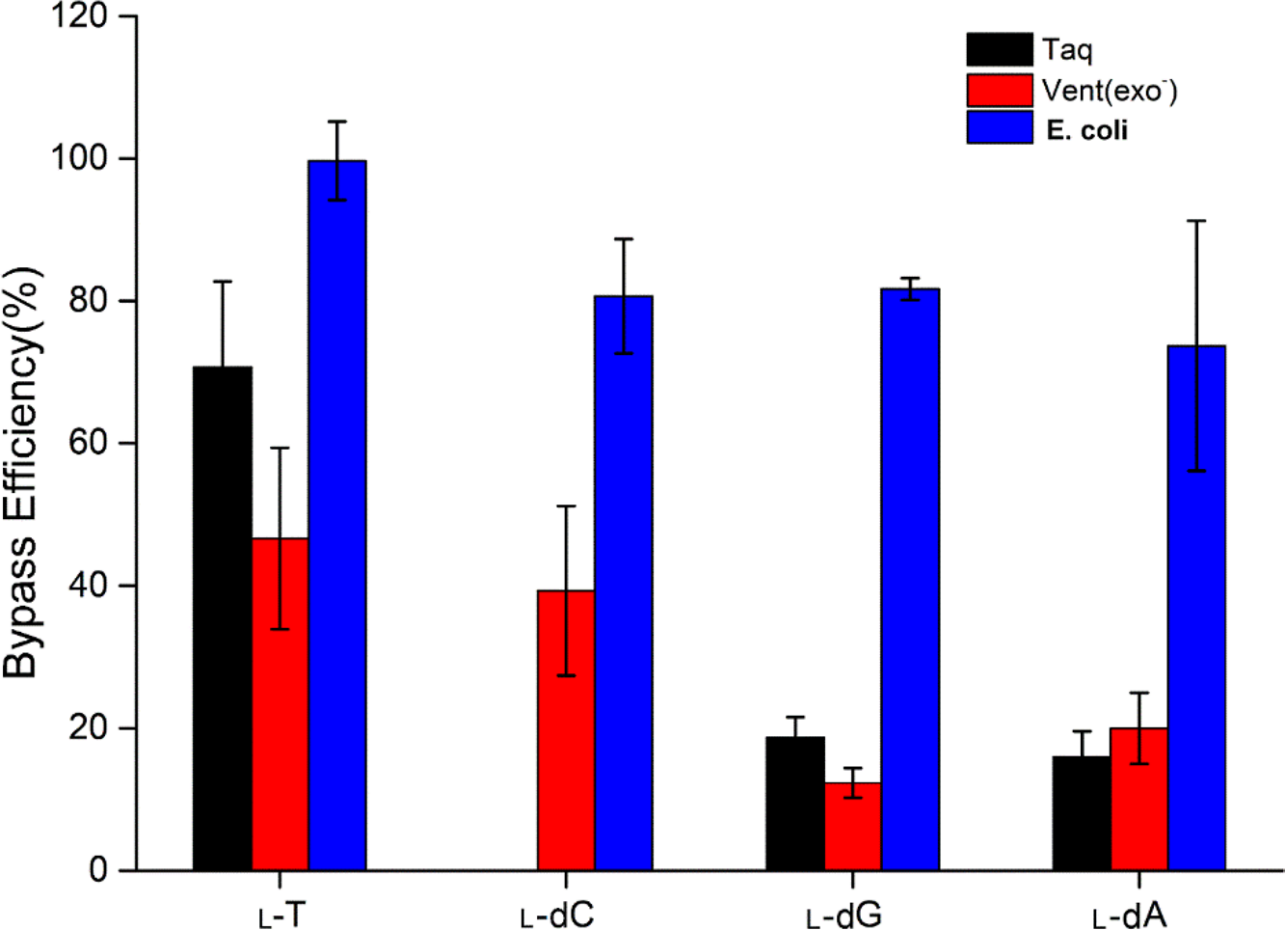
The bypass efficiency of replication of DNA containing l-nucleosites in cells and in vitro solution.

We examined previously the cytotoxic and mutagenic properties of l–T in *E. coli* as well as several DNA polymerases ^20,21^, which revealed distinctly different bypass efficiencies and mutagenic properties in different replication systems. This study was systematic interrogation about the impact of the l-nucleosides on the bypass efficiency and fidelity of DNA replication. Our results revealed that l-nucleosides could be readily bypassed by DNA replication machinery in *E. coli* cells (see Fig. 5 and Supplementary Table S2 online). Moreover, relative to Taq DNA polymerase and Vent (exo^-^) DNA polymerases, the relatively high efficiencies in bypassing l-nucleosides were observed in *E. coli* cells, where bypass efficiencies of l-dC, l-dG and l-dA being 81%, 82% and 74%, respectively. This finding paralleled the observations made in *E. coli* cells for l–T, which showed elevated bypass efficiency of l–T (to 99%). On the other hand, the study displayed the markedly decreased efficiency in bypassing l-nucleosides by Taq DNA polymerase and Vent (exo^-^) DNA polymerase for in vitro study and even completely inhibition for l-dC by Taq DNA polymerase, suggesting that l-nucleosides exerted a strong blockage effect on in vitro DNA replication. This difference of bypass efficiency in vitro and in cells could be associated with differences of *E. coli* polymerases and tested DNA polymerases, or cellular factors increased the bypass efficiency of l-nucleotides. *E. coli* replication system involved in five kinds of DNA polymerases (I, II, III, IV, V), and three of five DNA polymerases had high expression levels by the SOS DNA-damage response. Pol II was responsible for bacterial DNA repair and replication restart after DNA damage^24^. Pol III is an asymmetric, dimeric enzyme, which carries out the high-speed replication of the two DNA strands^22^. Pol IV and Pol V, which are Y-family DNA polymerases^24^, have low-processivity error-prone ability to bypass lesions via translesion synthesis. Similar to Y family polymerases in *E. coli*, some cellular factors, such as UmuD and UmuC, could involve in bypassing distinct template lesions^25^. Ogt, an alkyltransferase in *E. coli*, involved in repairing O4-alkyl dT with small straight-chain alkyl functionality^26^. What’s more, many enzymes showed synergism with each other^27^. Therefore, a tested DNA polymerase used in in vitro experiments has a special function, whereas the replication system in *E. coli* is so complicated that bypass efficiency of l-nucleotides on the template can be increased in *E. coli.*

For in vitro replication, Taq DNA polymerase and Vent (exo^-^) DNA polymerase exhibited similar effects on bypassing different l-nucleosides except l-dC. The observation underscored that l-dC completely inhibited Taq DNA polymerase, whereas it can be bypassed by Vent (exo^-^) DNA polymerases (Fig. 5). Thereby we performed molecular dynamics (MD) simulation to understand about the impact of l-nucleosides on DNA replication and to uncover the roles of DNA polymerases in bypassing these lesions. Considering that PDB crystallographic data for Vent (exo^-^) DNA polymerase was not available, we replaced Vent (exo^-^) DNA polymerase with Deep vent DNA polymerase, since they belonged to the B family of DNA polymerases and had the similar active domain.

For Taq DNA polymerase assemblies, root mean square deviation (RMSD) values of the complex containing l-dC, l-dG and l-dA modified duplexes as well as the normal DNA duplexes obtained from the final 10 ns simulations were presented in Supplementary Fig. S10a–c. l-dA and l-dG-contained duplexes showed RMSD values within the range of 0–5 Å, which were quite close to normal duplex (0–5.5). The l-dC-modified duplexes, by contrast, showed a high RMSD value (0–4.5) relative to normal conformation. Moreover, the results showed that the pseudorotation angles of l-dA and l-dG exhibited the same sugar conformation to regular conformation, associated to C2′-exo and C3′-endo conformation, whereas l-dC associated to O4′-exo-C1′-endo conformation type, resulting in a twist structure (see Supplementary Fig. S10d, e, f online). Together, these results reflected that l-dG and l-dA were bypassed by Taq DNA polymerase whereas l-dC was not.

In contrast to Taq DNA polymerase, experiment showed that all of the l-nucleosides were bypassed by Vent (exo^-^) DNA polymerase, although l-nucleosides moderately blocked DNA replication. This can be verified by the fact that RMSD values has no obvious difference in assemblies between l-dG, l-dA and l-dC and the corresponding D-nucleosides using Deep vent DNA polymerase (see Supplementary Fig. S11 online).

**Figure 6:**
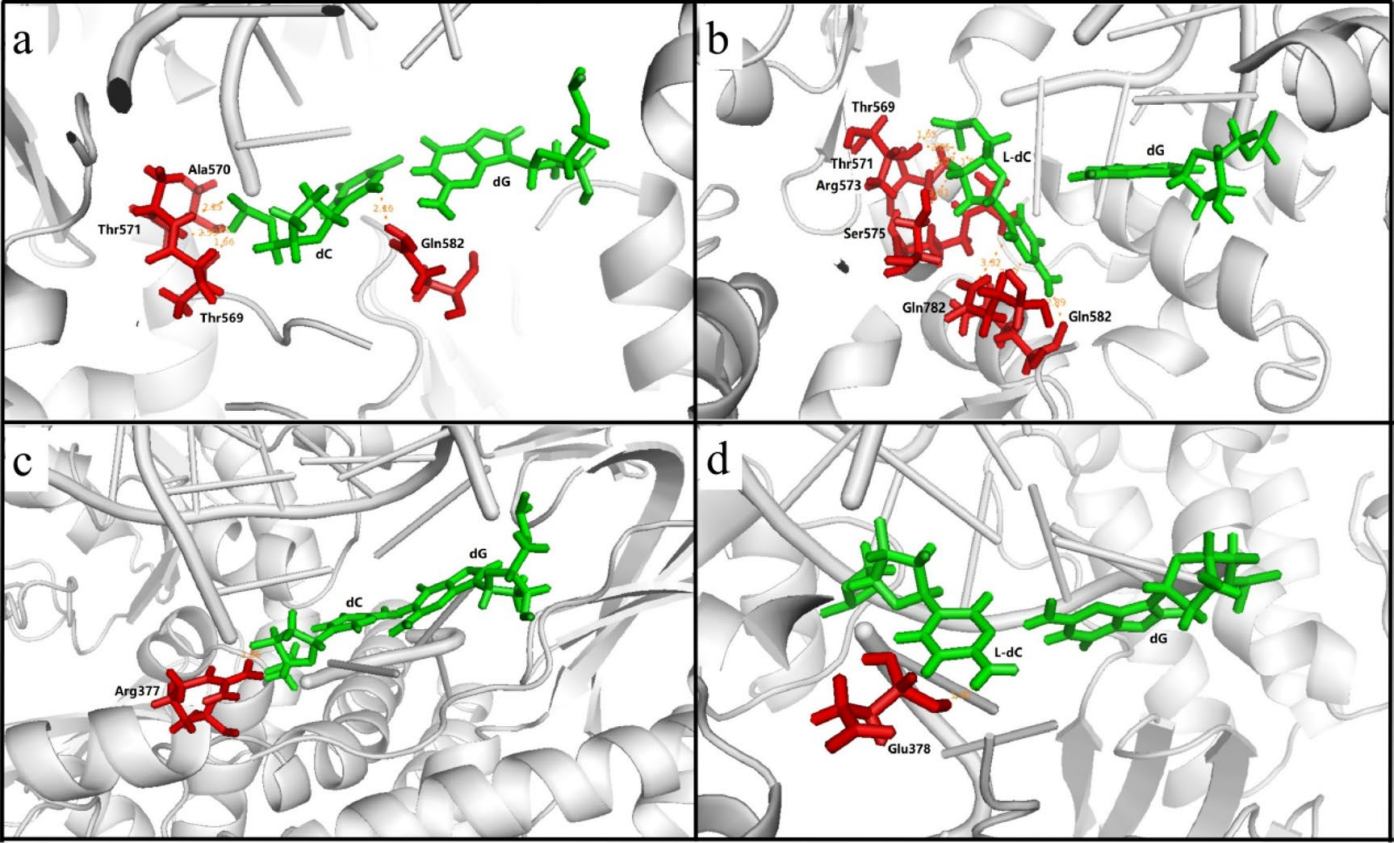
The cognate nucleotide insertion opposite to l-dC and dC by Taq DNA polymerase and Vent DNA polymerases. Taq DNA polymerase can bypass dC (**a**) but not bypass l-dC (**b**). Vent DNA polymerase can bypass not only dC (**c**) but also l-dC (**d**). Nucleotide pairs were shown in green, neighbouring amino acid residues were shown in red.

On the basis of these results, we reasoned that the polymerase played a key role in bypassing l-nucleoside lesions. It was known that family B DNA polymerases (Deep vent, Vent (exo^−^), and Therminator) were more efficient at incorporating chemical modifications in their substrates than the DNA polymerases from family A, such as Taq DNA polymerase^19,28–30^, because of striking difference derives from several key catalytic amino residues of O-helix of the finger domain^31^. As shown in Fig. 6, the nucleotide selection in Taq DNA polymerase was coordinated by this highly conserved residues Thr569 (O–H···O–P), Ala570 (N–H···O–P), Thr571 (O–H···O–P), Gln582 (N–H···O–C) in active domain (Fig. 6a). Nevertheless, when the template dC were replaced with l-dC, the key residues were arranged to residues Thr569 (O–H···O–P), Thr571 (O–H···O–P), Arg573 (N–H···O–P), Ser575 (O–H···O–C), Gln782 (N–H···O–C), Gln582 (N–H···O–C) (Fig. 6b), hydrogen bonded with l-dC to drag nucleotide at pre-translocation configuration, where hydrogen bonds on deoxyribose produce the main effect, such as Arg573 (N–H···O-P), Ser575 (O–H···O–C), Gln782 (N–H···O–C), converting favourable 3′-endo-sugar conformation to unfavourable O4′-exo-C1′-endo conformation, resulting in enzyme's failure to bypass of the lesion. For Deep vent DNA polymerase assemblies, the critical catalytic residues may be rearranged from Arg377 (N–H···O–C) to Glu378 (N–H···O–C) (Fig. 6c, d) when the template base switched from dC to l-dC. Obviously, the sterical clash with the side chain of amino acid residue in Deep vent DNA polymerase can be much reduced relative to Taq DNA polymerase, without the conformation and hydrogen bonding force being changed, thereby Deep vent DNA polymerase was also capable of incorporating nucleotides opposite to l-dC. In addition, when the template base changed from dC to l-dC in the complexes of Taq polymerase, and the binding energy changes from − 341.6501 to − 312.2146 (kcal/mol), producing an energy difference of 29.4355 (kcal/mol). In the Deep vent DNA polymerase, the binding energy changes from − 43.5818 to − 36.5313 (kcal/mol), producing an energy difference of 7.0505 (kcal/mol). Thus, the difference between l-dC and dC with respect to binding energy of the complexes of Taq polymerase was more pronounced than those of Deep vent DNA polymerase (see Supplementary Table S3 online).

Another major goal of the study was to examine the impact of the l-nucleoside lesions on fidelity of DNA replication. Although some DNA polymerases can employ damaged DNA as a template, nucleotide insertions mediated by these polymerases in cells were sometimes inaccurate, thereby leading to mutations^32^. Previous results revealed that l–T was found not to be mutagenic by a single DNA polymerase ^19^, whereas it displayed mutagenic effects in *E. coli* cells, where TLS polymerases may have different mutagenic affects in extending the nascent strand from l–T-containing strand^21^. In the study, l-dC induced no mutation on DNA replication in vitro solution and in cells (Fig. 2d). Interestingly, l-dG lesion was no mutagenic in cells, whereas l-dG → T and l-dG → dG conversions catalysed by Vent (exo^-^) DNA polymerase was observed, where l-dG → T frequency (65%) was two times higher than l-dG → dG frequency (see Fig. 3d and Supplementary Table S1 online). Our results also demonstrated that l-dA exhibited the l-dA → T (37%) and l-dA → dA (63%) conversions for Taq DNA polymerase and l-dA → T (100%) conversions for Vent (exo^-^) DNA polymerase. Differently, l-dA also induced more types of conversions in cell replication, i.e. l-dA → dA (67%), l-dA → dG (24%) and l-dA → dC (9%) (see Fig. 4d and Supplementary Table S1 online). Among the four l-nucleoside lesions, l–T exhibited the highest frequency of nucleoside misincorporation in *E. coli* cells, with l–T → dA (13%), l–T → dG (19%) and l–T → dC (46%) mutations (Supplementary Table S1 and Fig. S12 online). The difference in mutagenic frequencies of l-nucleosides bypass in vivo and in vitro may be attributed to functional interaction between TLS polymerases and other related proteins^33–35^.

**Figure 7:**
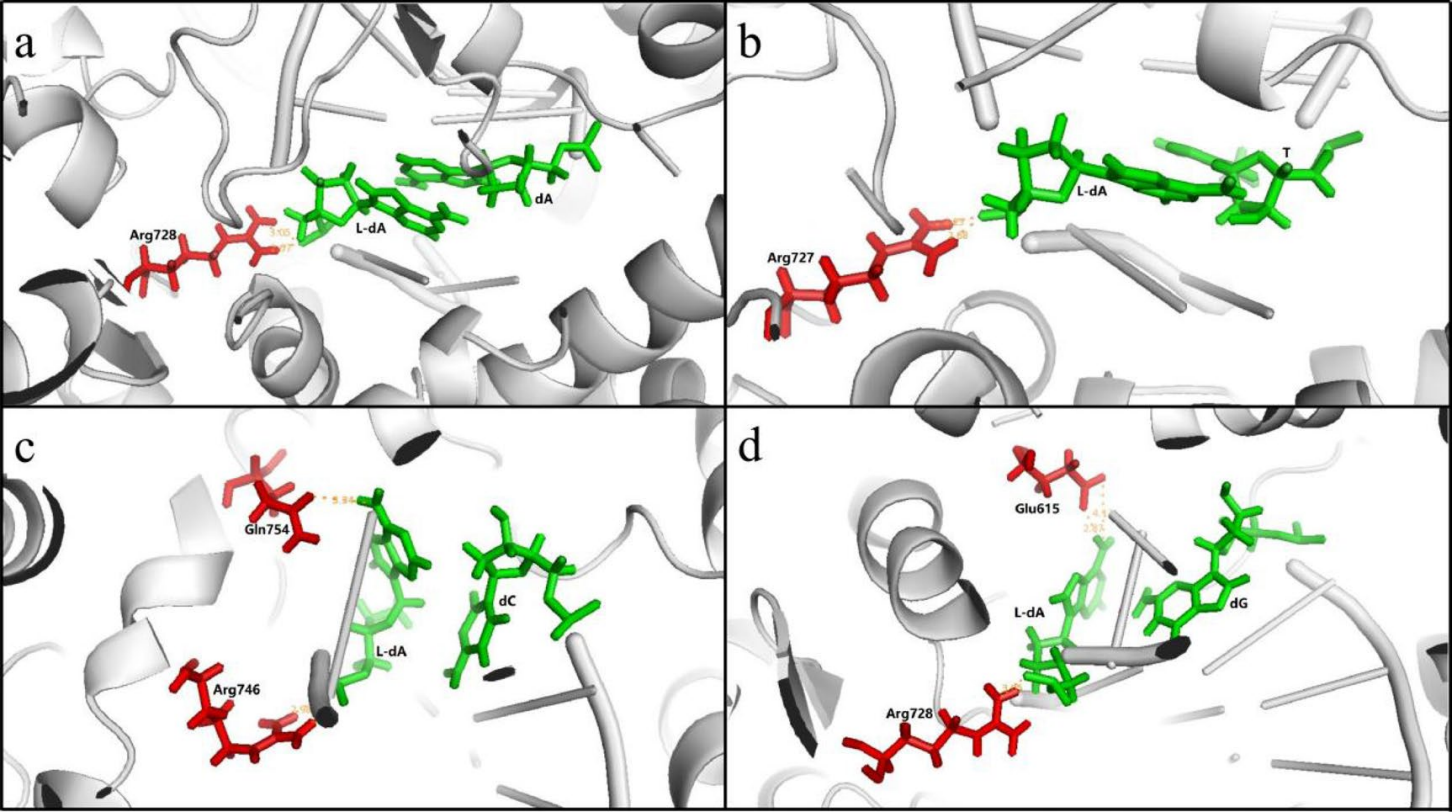
The cognate and non-cognate nucleotide insertion opposite to l-dA by Taq DNA polymerase. (**a**) l-dA:dA, (**b**) l-dA:T, (**c**) l-dA:dC, (**d**) l-dA:dG. Nucleotide pairs were shown in green, neighbouring amino acid residues were shown in red.

Except that DNA polymerases have relative low error rate on replication, the mutagenic effect may attribute to change of the stereochemistry of the templating nucleotide from D to L, which was expected to alter its interactions with the incoming nucleotide and active site residues and influence nucleotide binding and incorporation^36^. Therefore, we performed molecular simulations to investigate how these lesions elicited mutations. We performed MD simulation for insertion of the cognate and non-cognate nucleotides opposite to l-nucleosides by using a template strand containing an l-deoxynucleoside to pair with the primer strand with the insertion of a dNTP in the presence of DNA polymerase. The results revealed that whether l-nucleosides elicited mutations depended on the interaction of the base pair formed from l-nucleosides and the incoming nucleotide with active site residues despite of ribosyl alteration from D to L. Figure 7 and Supplementary Fig. S13 showed this vein related to Taq DNA polymerase involved in nucleotide incorporation opposite l-dA. In the normal case, the residue Arg728 of O-helix interacts with dA the template (Fig. 7a) to operate dNTP by conformational changes from an “open” complex to a catalytically competent “closed” complex^37^. When l-dA was introduced into the template, the key catalytic residues Arg727 (N–H···O–P), Arg728 (N–H···O–P), hydrogen bonded to l-dA, can pack against the nascent base pair l-dA:T and l-dA:dA, respectively, forming closed ternary complexes, similar to the key residues of dA:T (Fig. 7a, b and Supplementary Fig. S13). Differently, l-dA in the nascent base pair l-dA: dC and l-dA: dG was confined to two key residues Arg746 (N–H···O–P) and Gln754 (N–H···O–C) for l-dA: dC, Arg728 (N–H···O–P) and Gln615 (N–H···O–C) for l-dA: dG by hydrogen bonds, displayed a twist structure deviated from Watson–Crick base pairs. When the l-dA alignment is dC or dG, l-dA is constrained by hydrogen bonds between the hydroxyl and phosphate oxygen. As a result, the closed conformations can be not stabilized by these interactions. Thus, Taq polymerase has a tendency to incorporate dATP and dTTP, but no tendency to insert dGTP and dCTP (see Fig. 7 and Supplementary Table S1 online). Besides, the topological difference of more DNA polymerases would be needed to reveal whether this was the case by analysis of the X-ray crystal structure of their complexes in the future.

## Conclusions

In conclusions, our systematic study based on shuttle vector that containing l-nucleosides provided important novel insights about effect of l-nucleosides on efficiency and fidelity of DNA replication in vitro and in *E. coli* cells. Our results indicated that l-nucleosides exhibited pronounced replicative efficiency in bypassing the lesions in *E. coli* cells, whereas they exerted a strong blockage effect on DNA replication by Taq DNA polymerases or Vent (exo^-^) DNA polymerases, and especially l-dC completely inhibited DNA replication by Taq DNA polymerases. Additionally, we found that l-dA and l–T stimulated various types of mismatch in *E. coli* cells, including purine → pyrimidine or pyrimidine → purine conversion and purine → purine or pyrimidine → pyrimidine transversion. MD simulations were performed to clarify the change in structures, dynamics, stability of DNA-polymerase assemblies with incorporation of l-nucleosides, and help to understand how l-nucleosides impair the efficiency and fidelity of DNA replication. Further experiments will be needed to investigate the repair pathway and mechanism due to the effect of multiple family DNA polymerases. Our study provides a new understanding of the effect of l-2′-deoxynucleosides on DNA replication in vitro solution and in cells.

## Material and methods

Plasmid pUC19, DH5α competent cells, Taq DNA polymerase, X-gal, and dNTPs were purchased from Tiangen Biotec Co., Ltd. (Beijing, China). Vent (exo^-^) DNA polymerase and restriction enzymes (*Hind*III, *EcoR*I, and *Pst*I) were obtained from New England Biolabs Inc (Ipswich, MA, USA). SYBR® gold nucleic acid gel stain was purchased from Invitrogen, Ltd. (Paisley, UK). T4 DNA ligase and T4 polynucleotide kinase were purchased from Beyotime (Shanghai, China). Plasmid small-extraction kit and 40% acrylamide (acrylamide:bis-acrylamide 19:1) were from Beijing Solarbio Science & Technology Co., Ltd. (Beijing, China). The E.Z.N.A.® Cycle-Pure Kit was obtained from Omega Biotek Inc (Georgia, USA). All oligonucleotides used in this study were purchased from Suzhou Beixin Biotechnology Co., Ltd. Ultrapure water was prepared using double distilled water (UPW, 18 MΩ·cm).

The following equipments were used: PAGE electrophoresis system (Junyi JY200C, Beijing, China), Micro high-speed centrifuge (Xiangyi TG16-W, Changsha, China), Microvolume UV–Vis spectrophotometer (NanoDrop2000 Thermo Fisher, Boston, Massachusetts, USA), ChemiDoc™ MP Imaging System (BioRad, Hercules, California, USA), MJ MiNi Thermal Cycler (Bio-Rad PTC-1148, Hercules, California, USA), and High-speed refrigerated centrifuge (Xiangyi TGL-16, Changsha, China).

### Preparation of the **l**-nucleoside-carrying 24-mer ODNs

20 μL of reactions that contains 2 μL of reaction buffer A (10 $\times$), an equimolar amount of l-nucleoside-carrying ODNs (i.e. G13 containing l-dG,$5{^\prime}\hbox{-AATTCTC}\underline{{\rm CG}{^{\rm L}}{\rm GCAG}}\hbox{GCTAGCACTGA-}3{^\prime}$) and its complementary strand (COM20, 5′-AGCTTCAGTGCTAGCCTGCACGAG-3′), 1 μL of 0.1 mM ATP, 1 μL of T4 polynucleotide kinase (10 U/μL), and nuclease-free deionized water were mixed thoroughly and 5′- phosphorylated at 37 °C for 30 min, followed by incubation at 95 °C for 10 min and cooling for 10 min at room temperature.

### Construction of **l**-nucleosides-containing genomes

According to the previously reported procedures^21^, 300 ng of pUC19 plasmids were digestion with 20 μL *EcoR*I (20000U mL^−1^) and 20 μL *Hind*III (20000U mL^−1^). The resulting linearized vector was isolated by E.Z.N.A.® Cycle-Pure Kit, following the manufacturer′s instructions. The ligation reactions in 20 μL of reaction containing the linearized vector and double-stranded insert at a molar ratio of vector to insert of 1:3, 2 μL of 10 × T4 DNA ligase buffer, 0.5 μL (1000U μL) of T4 DNA ligase, and distilled water, were performed at 16 °C for overnight.

### Transfection of **l**-nucleosides-containing genomes

Prior to plasmid-containing cell culture, 10 μL of ligation mixture was transformed into 50 μL chemically competent DH5α *E. coli* cells by heat shock at 42 °C for 90 s. Then, 400 μL LB liquid medium was added into above cells, followed by cultivation at 37 °C with shaking (225 rpm) for 1 h. lastly, 300 μL cultured bacterial can be added into another 5 mL LB liquid medium containing AMP (ampicillin) at concentration of 100 mg/L and cultivated at 37 °C with shaking (225 rpm) for 14–16 h. The progeny genomes were isolated using plasmid small-extraction kit.

### Quantification of bypass efficiencies and mutation frequencies

The version of restriction enzyme–mediated assay developed by us was employed to assess the bypass efficiencies and mutation frequency of the DNA lesion in vitro and in cells^20^ (Fig. 1). The l-nucleoside-carrying recombinants from ligation reaction and progeny genomes arising from cellular replication, representing as in vitro and in cell replication study, were amplified by PCR with the use of Taq DNA polymerase, respectively. The two primers were C12F 5′-TTAGCTCACTCATTAGGCACCC-3′ and C12R 5′-GGGCCTCTTCGCTATTACGC-3′, and the PCR amplifications initiated at 95 °C for 3 min, followed by 39 cycles at 95 °C for 30 s, 52.2 °C for 30 s, and 72 °C for 1 min, a final extension at 72 °C for 5 min, and hold at 4 °C. The PCR products were purified using Cycle Pure Kit (Omega) and stored at − 20 °C until use.

For the analysis of bypass efficiency, a portion of the PCR products was treated with *EcoR*I/*Hind*III and *Pst*I/*Hind*III at 37 °C in 5 μL of NEB CutSmart buffer for 4 h, respectively, referring to NEB restriction digest protocol. The reaction mixture was quenching with 3 (for *EcoR*I/*Hind*III digestion products) and 1 (for *Pst*I/*Hind*III digestion products) time volume of 2 × denaturing DNA loading buffer containing bromophenol blue dyes. The mixture was loaded onto 20% denature polyacrylamide gel (acrylamide : bis-acrylamide = 19:1) and electrophoresis was done at 150 V for 1.5 h. The gel was stained with SYBR Gold Nucleic Acid Gel Stain and photographed using a ChemiDoc™ MP Imaging System (Bio-Rad). The quantification of band intensities was conducted using Image Lab software version 6.0 (Bio-Rad).

The effects of l-nucleosides on DNA replication were determined by the relative bypass efficiency (RBE) and mutation frequency values, respectively. The mutation frequency was calculated from the percentage of mutagenic product (i.e. the product with l-G → T mutation) among the sum of all products arising from replication of the lesion-containing genome. The bypass efficiency was sum of all relative incorporation efficiency of all nucleotides opposite l-nucleosides.

### Molecular dynamics simulations

The crystal structures of the pre-translocation (closed) state of Taq DNA polymerase (3KTQ) ^38^ and the pre-translocation (closed) state of Deep vent DNA polymerase (5H12) ^39^ both obtained using X-ray diffraction, were downloaded from the PDB crystal library. The Taq DNA polymerase was in the pre-translocation state, where the finger structure domain was in the closed state and the C-terminal position of the O-helix was in the "Pre" state. Deep vent DNA polymerase with structureless DNA requires software to bind to the DNA duplex under study and then pre-process the two complex structures accordingly.

This part of the study used the PMEMD software ^40^ in Amber 16 software on a GPU platform to run the kinetic simulations. Before the kinetic simulations, the complex system was hydrogenated using the leap program. The force field used for the protein was the ff14SB force field ^41^, and the force field used for DNA was the DNA. OL15 ^42^ force field. The complexes were then placed in a 12 Å positive chipped octahedral box of aqueous solvent, and the water molecules were made electrically neutral by using TIP3P ^43^, which added counterbalancing ions (Na^+^ ,Cl^−^) to the entire complex system.

After processing the complex system using the leap procedure, the whole system was optimized. After optimization, two ramp-up processes were carried out for the complexes within the NVT system in seven steps, from 0 to 310 K and 345.15 K. After optimization and ramp-up, molecular dynamics simulations were carried out for each complex for 10 ns, with a time step of 2 fs and a frame saved at 1 ps intervals. The SHAKE algorithm was used to control the stretching of the chemical bonds containing the nitrogen atoms and the truncation value of the non-bonded interactions was set to 10 Å.

The cpptraj program in Amber 16 was used for the analysis of trajectories, the analysis of RMSD, hydrogen bonding analysis, and distance analysis. This part of study used the MM/GBSA method to calculate the interaction energy of DNA with proteins. PYMOL^44^, Origin and MATLAB were used for graphing and data analysis.

## Supplementary Information

Supplementary Information.

## Data Availability

The datasets generated during and/or analysed during the current study are available from the corresponding author on reasonable request.
