# Supplementary material for: Replicative bypass studies of l-deoxyribonucleosides in Vitro and in E. coli cell
Source: Sci Rep. 2022 Dec 7;12:21183. doi: 10.1038/s41598-022-24802-5 (PMC9729220; doi:10.1038/s41598-022-24802-5)
Supplement: Supplementary file 1 — Supplementary Information. [file 41598_2022_24802_MOESM1_ESM.pdf]

Supporting Information for

Replicative Bypass Studies of L-deoxyribonucleosides *in Vitro* and in *E. coli* cell

Yuhe Kan[1,4,5†], Zhaoyang Jin[1†], Yongqi Ke[1], Dao Lin[1], Liang Yan[1], Li Wu[1,2*] & Yujian He[1,2,3*]

[1] School of Chemical Sciences, University of Chinese Academy of Sciences, Beijing 100049, PR China
[2] State Key Laboratory of Natural and Biomimetic Drugs, School of Pharmaceutical Sciences, Peking University, Beijing 100191, PR China.
[3] School of Future Technology, University of Chinese Academy of Sciences, Beijing 100049, PR China
[4] School of Life Sciences, Inner Mongolia University, Hohhot 010021, Inner Mongolia, PR China
[5] Qilu Pharmaceutical (Inner Mongolia) CO., LTD., Hohhot 010080, Inner Mongolia, PR China
[*]Correspondence and requests for materials should be addressed to L.W. (e-mail: wuli@ucas.ac.cn) and Y.J.H. (e-mail: heyujian@ucas.ac.cn)
[†]these authors contributed equally to this work

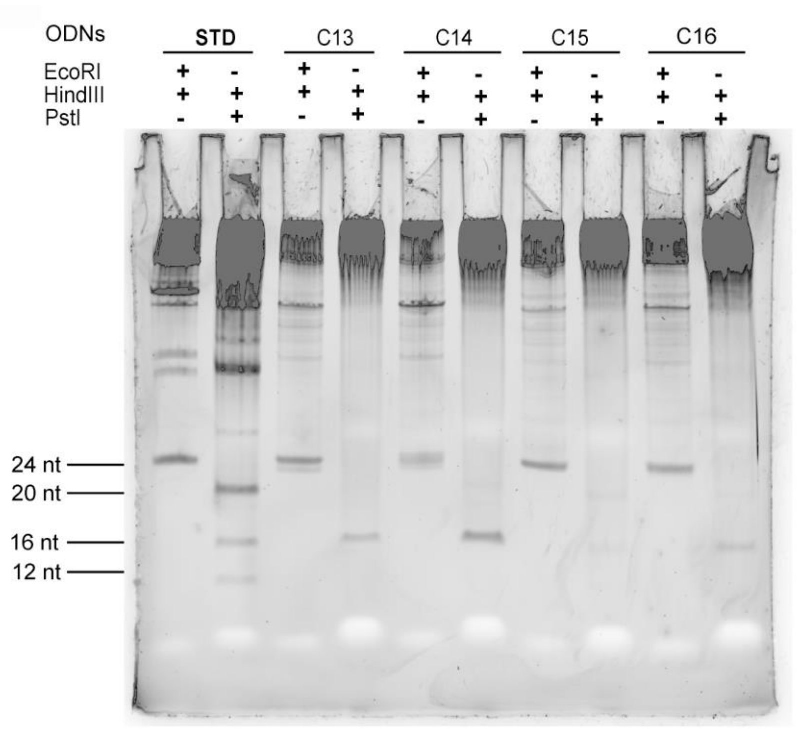

Figure S1. PAGE analysis of restriction fragments of replication products arising from the L-dC lesion-bearing genome under the catalysis of Taq DNA polymerase.

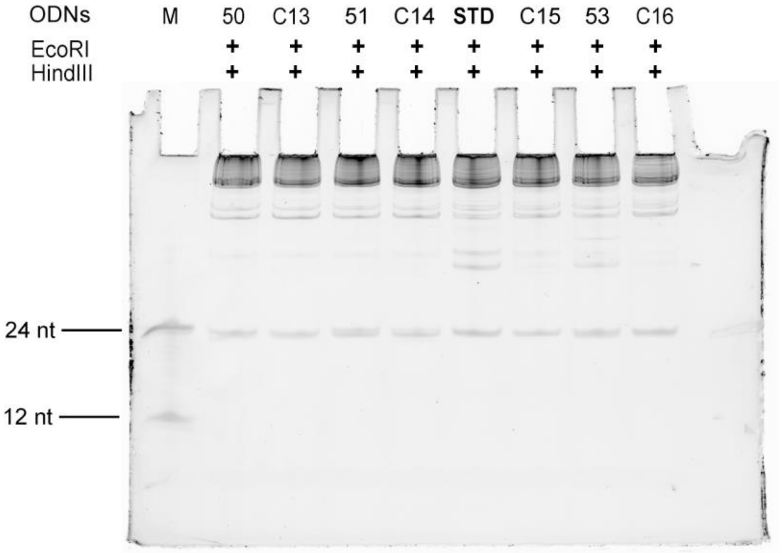

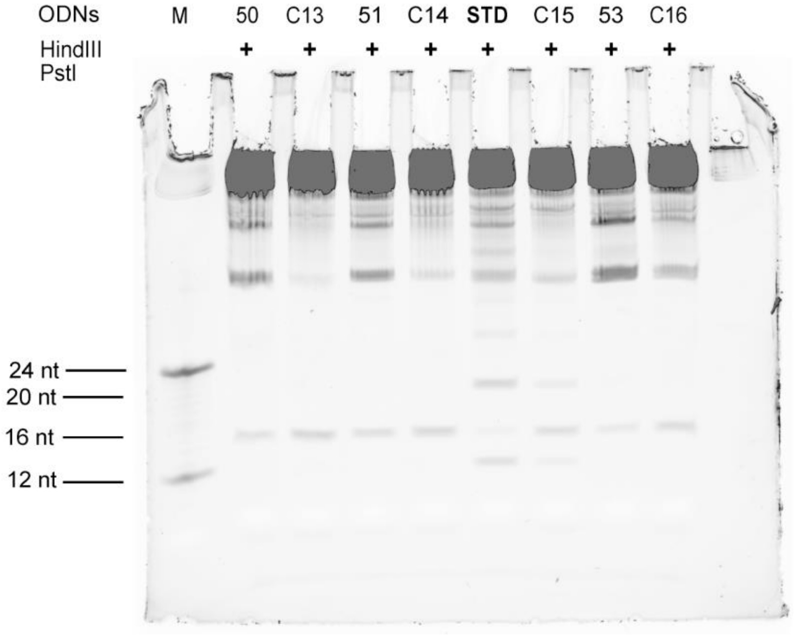

Figure S2. PAGE analysis of restriction fragments of replication products arising from the L-dC lesion-bearing genome under the catalysis of Vent (exo^{-}) DNA polymerase.

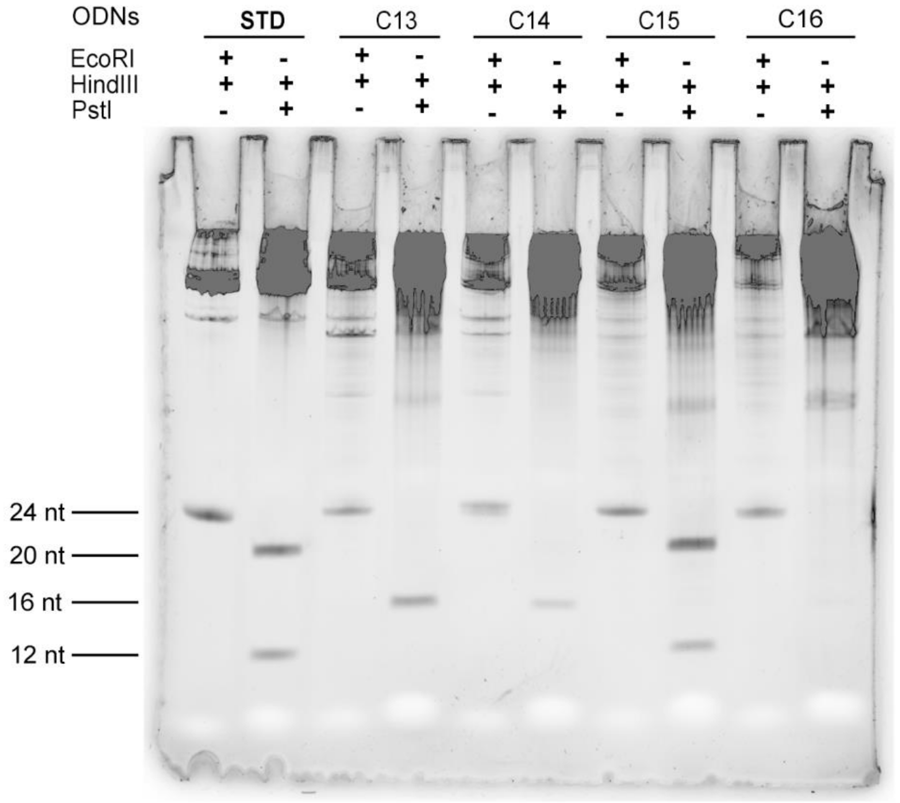

Figure S3. PAGE analysis of restriction fragments of replication products arising from the L-dC lesion-bearing genome in *E. coli* cells.

		→T (%)	→dG (%)	→dC (%)	→dA (%)	Bypass efficiency (%)/ Incorporation frequency (%)
Taq DNA polymerase	L -T	71	—	—	—	71
		100	—	—	—	100
	L-dC	—	—	—	—	—
		—	—	—	—	—
	L-dG	—	19	—	—	19
		—	100	—	—	100
	L-dA	6	—	—	10	16
		37	—	—	63	100
Vent (exo⁻) DNA polymerase	L-T	47	—	—	—	47
		100	—	—	—	100
	L-dC	—	—	39	—	39
		—	—	100	—	100
	L-dG	8	4	—	—	12
		65	35	—	—	100
	L-dA	20	—	—	—	20
		100	—	—	—	100
E. coli cell	L-T	22	19	45	13	99
		22	19	46	13	100
	L-dC	—	—	81	—	81
		—	—	100	—	100
	L-dG	—	82	—	—	82
		—	100	—	—	100
	L-dA	—	18	7	49	74
		—	24	9	67	100

Table S1. Incorporation frequency and bypass efficiency of replication of DNA containing L-2′-deoxynucleosides in different replication systems. The data represent the mean from three replicate experiments.

		In vitro solution				In cells	
		Taq DNA polymerase		Vent (exo⁻) DNA polymerase		*E. coli.* cell	
		Bypass Efficiency (%)	Whether Mutation	Bypass Efficiency (%)	Whether Mutation	Bypass Efficiency (%)	Whether Mutation
Pyrimidine	L-T	71	NO	47	NO	99	YES
	L-dC	0	NO	39	NO	81	NO
Purine	L-dG	19	NO	12	YES	82	NO
	L-dA	16	YES	20	YES	74	YES

Table S2. Replication of DNA containing L-2′-deoxynucleosides. The data represent the mean from three replicate experiments.

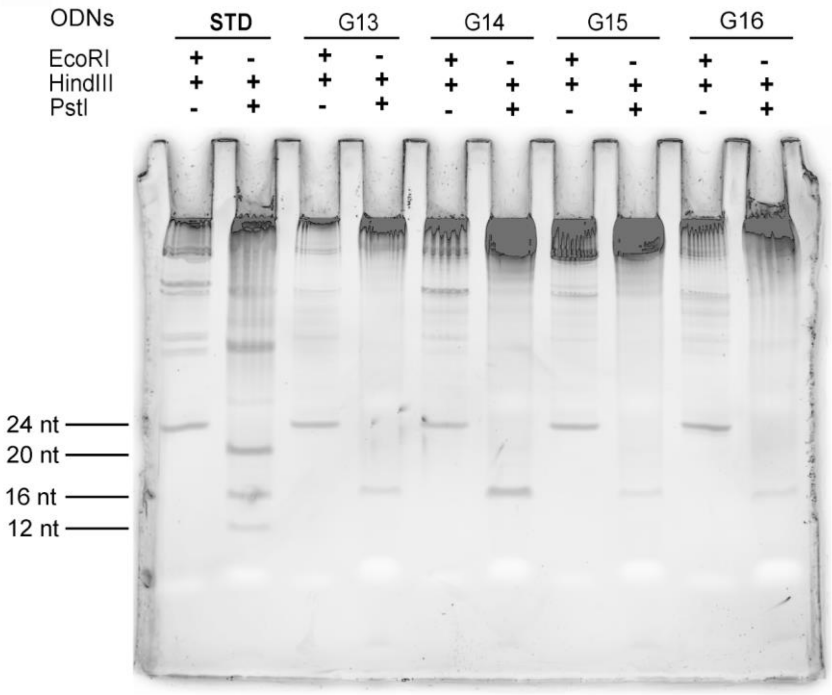

Figure S4. PAGE analysis of restriction fragments of replication products arising from the L-dG lesion-bearing genome under the catalysis of Taq DNA polymerase.

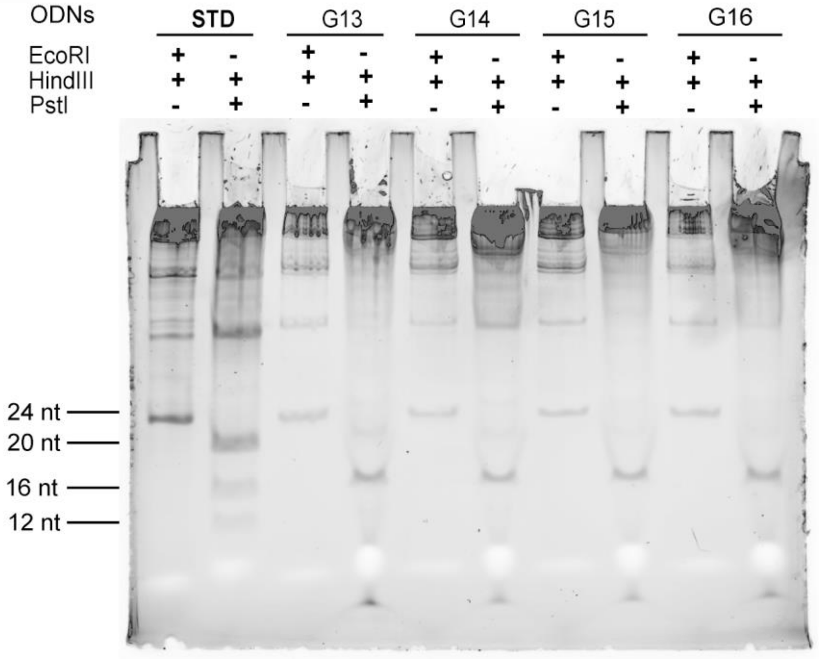

Figure S5. PAGE analysis of restriction fragments of replication products arising from the L-dG lesion-bearing genome under the catalysis of Vent (exo$^-$) DNA polymerase.

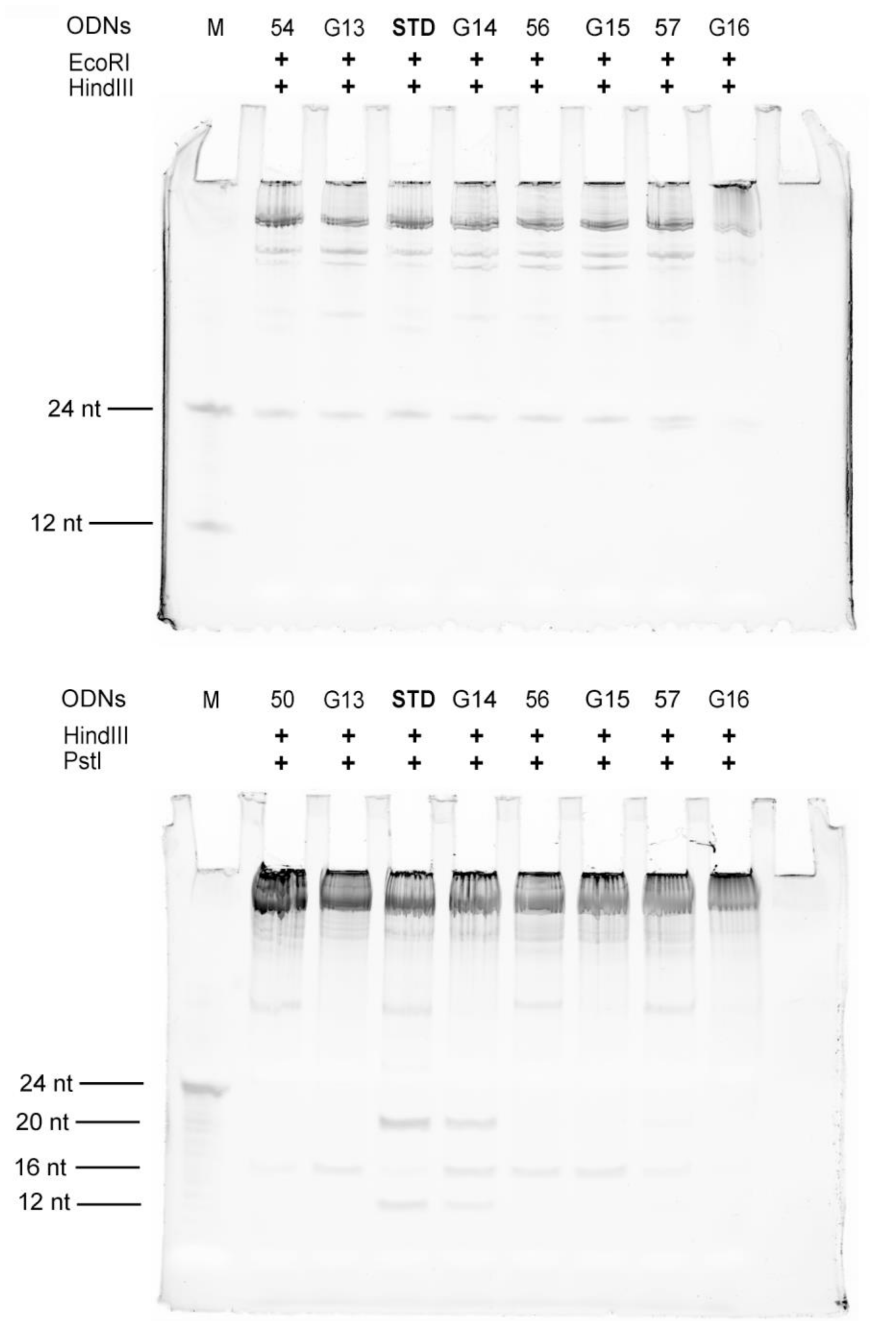

Figure S6. PAGE analysis of restriction fragments of replication products arising from the L-dG lesion-bearing genome in *E. coli* cells.

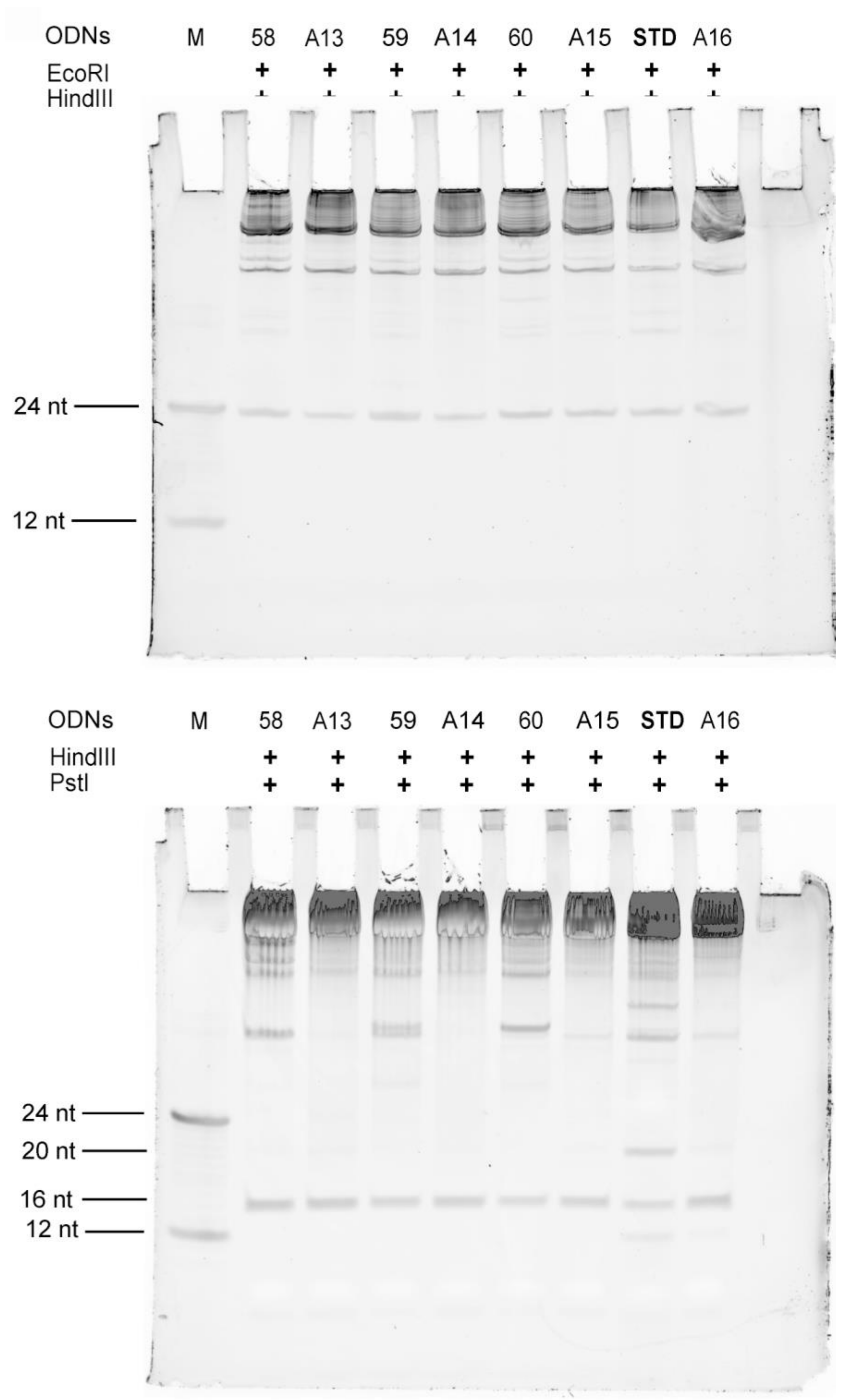

Figure S7. PAGE analysis of restriction fragments of replication products arising from the L-dA lesion-bearing genome under the catalysis of Taq DNA polymerase.

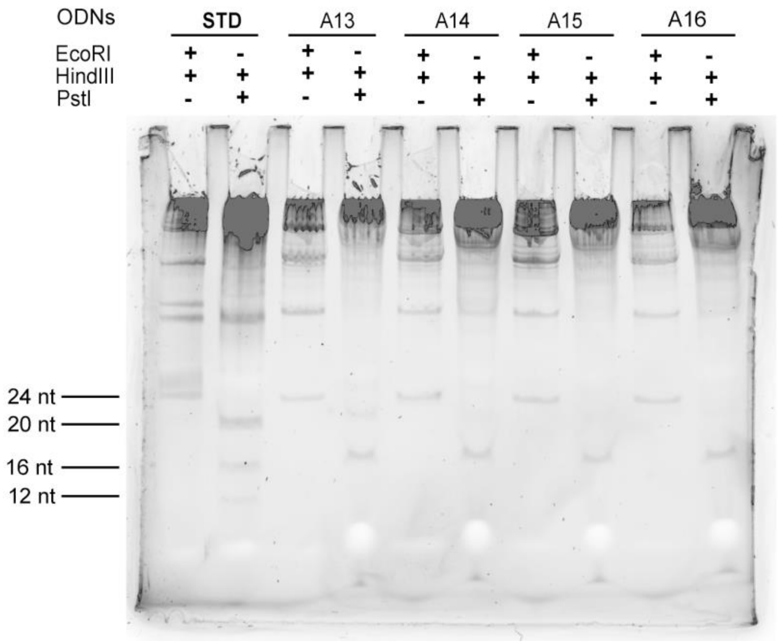

Figure S8. PAGE analysis of restriction fragments of replication products arising from the L-dA lesion-bearing genome under the catalysis of Vent (exo$^-$) DNA polymerase.

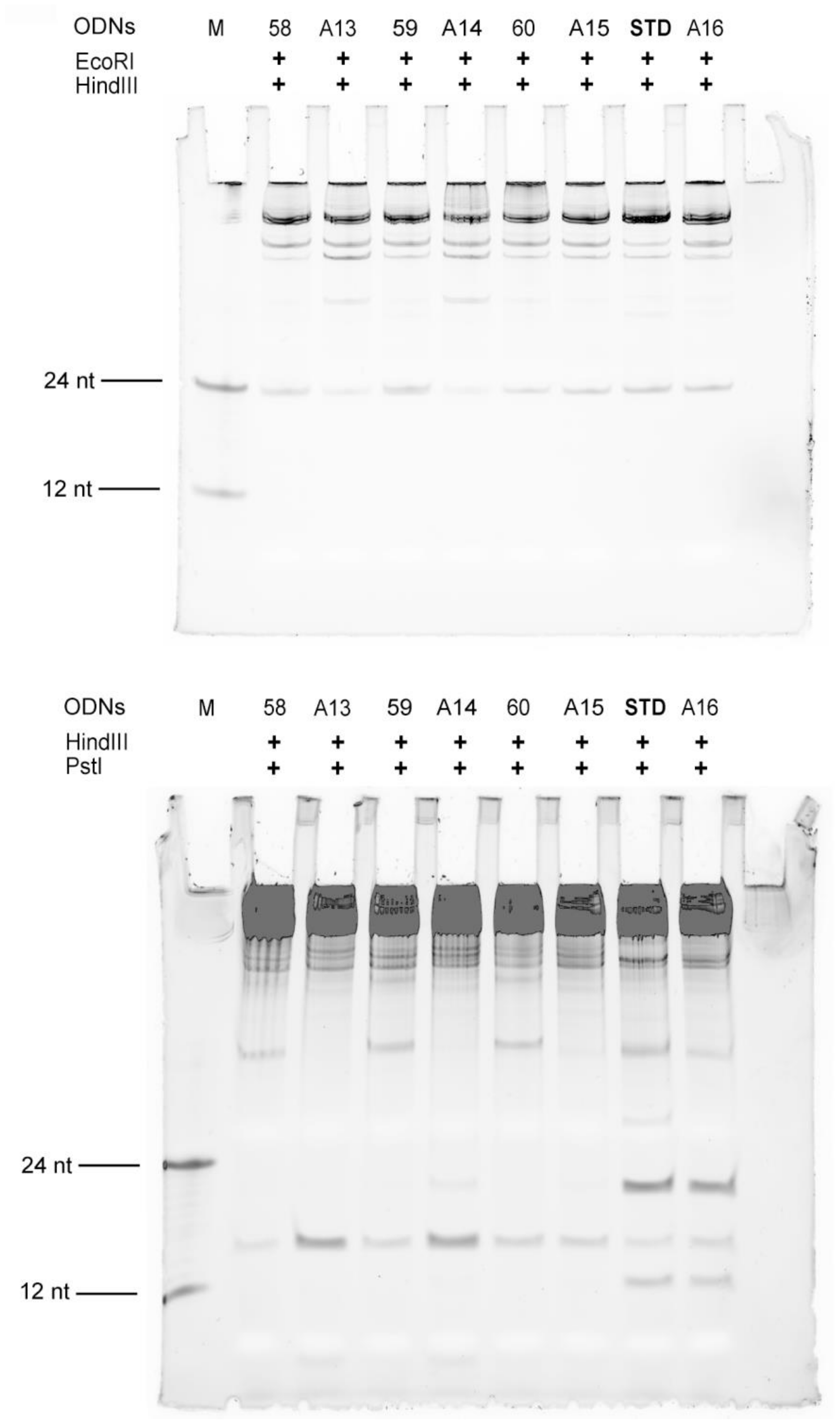

Figure S9. PAGE analysis of restriction fragments of replication products arising from the L-dA lesion-bearing genome in *E. coli* cells.

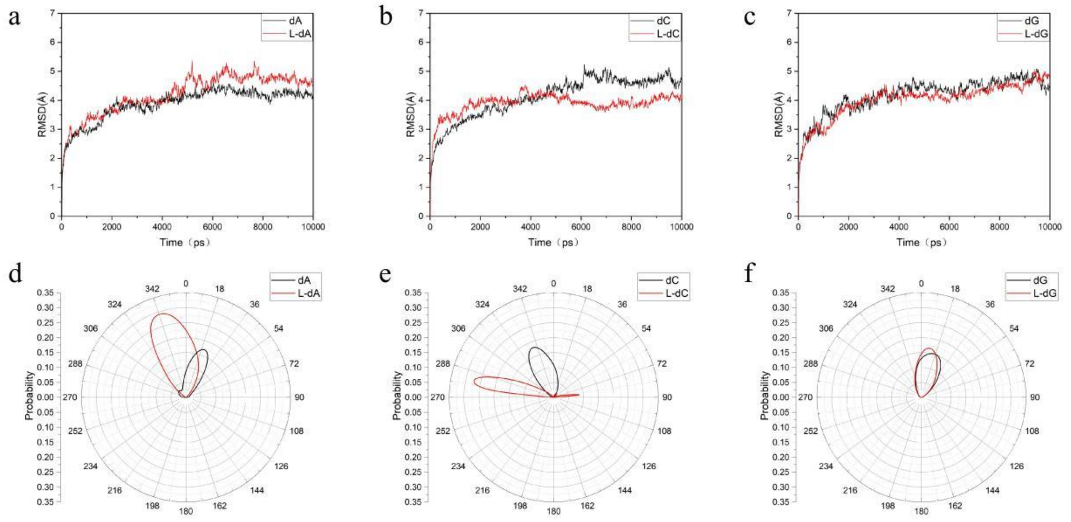

Figure S10. Comparison of (a) and (d) L-dA and dA, (b) and (e) L-dC and dC, and (c) and (f) L-dG and dG based on RMSD values and puckering angles by Taq DNA polymerase. (a)-(c) and (d)-(f) corresponds to RMSD values and puckering angles, respectively. Nucleotide pairs were shown in green, neighbouring amino acid residues were shown in red.

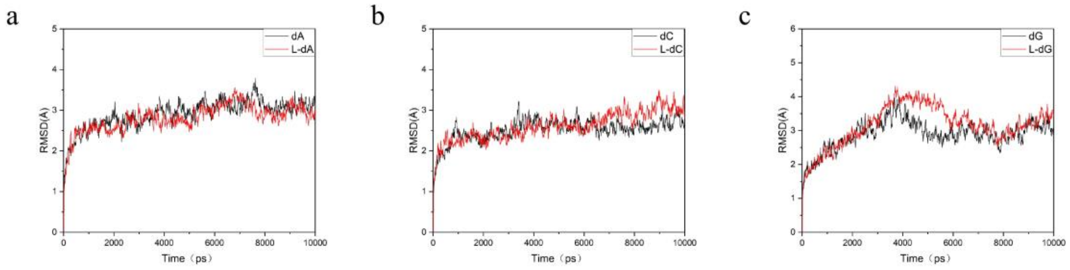

Figure S11. Comparison of (a) L-dA and dA, (b) L-dC and dC, and (c) L-dG and dG based on RMSD values in Deep vent DNA polymerase.

		Non-Polar Energy (kcal/mol)	Polar Energy (kcal/mol)	Binding Energy (kcal/mol)	Energy difference (kcal/mol)
Deep vent	dC	-149.8749	106.2931	-43.5818	7.0505
	L-dC	-163.3916	126.8603	-36.5313	
Taq	dC	-239.136	-102.5141	-341.6501	29.4355
	L-dC	-237.2395	-74.9751	-312.2146	

Table S3. The binding free energies of different polymerase assemblies

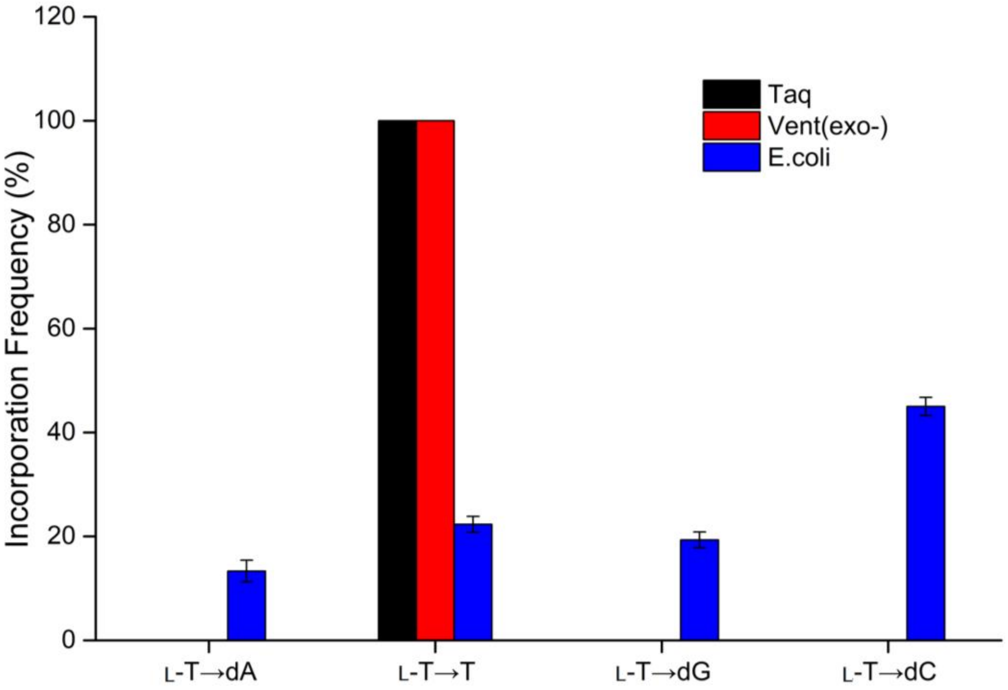

Figure S12. The mutation frequency of replication of DNA containing (a) L-dC, (b) L-dG, (c) L-dA and (d) L-T *in vitro* solution and in cells.

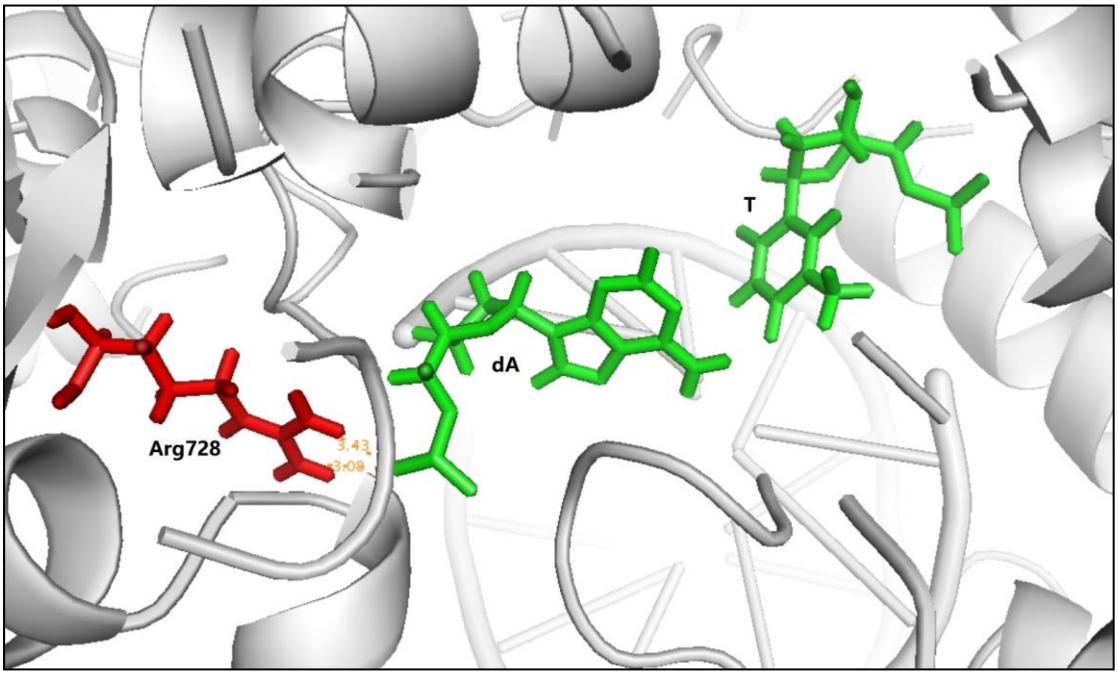

Figure S13. The cognate nucleotide insertion opposite to dA by Taq DNA polymerase. Nucleotide pairs were shown in green, neighbouring amino acid residues were shown in red.
